# Acyl-1,4-Dihydropyridines: Universal Acylation Reagents for Organic Synthesis

**DOI:** 10.3390/molecules29163844

**Published:** 2024-08-13

**Authors:** Karthikeyan Manoharan, Bartosz Bieszczad

**Affiliations:** 1 Centre for Synthesis and Chemical Biology, School of Chemistry, University College Dublin, Belfield, D04 V1W8 Dublin, Ireland; 2 School of Chemical and Pharmaceutical Sciences, Technological University Dublin, City Campus, Grangegorman, D07 H6K8 Dublin, Ireland

**Keywords:** dihydropyridines, acylation, acyl radicals, organic synthesis

## Abstract

Acyl-1,4-dihydropyridines have recently emerged as universal acylation reagents. These easy-to-make and bench-stable NADH biomimetics play the dual role of single-electron reductants and sources of acyl radicals. This review article discusses applications of acyl-1,4-dihydropyridines in organic synthesis since their introduction in 2019. Acyl-1,4-dihydropyridines, activated by photochemical, thermal or electrochemical methods, have been successfully applied as radical sources in multiple diverse organic transformations such as acyl radical addition to olefins, alkynes, imines and other acceptors, as well as in the late-stage functionalisation of natural products and APIs. Release of acyl radicals and an electron can be performed under mild conditions—in green solvents, under air and sunlight, and without the use of photocatalysts, photosensitizers or external oxidants—which makes them ideal reagents for organic chemists.

## 1. Introduction

**Scheme 1:**
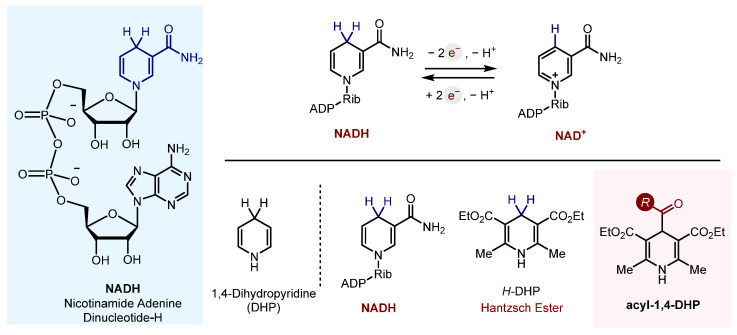
Dihydropyridines as synthetic equivalents of NADH.

Although it has been more than 140 years since Arthur Hantzsch reported the first synthesis of dihydropyridines (DHPs), new applications of these molecules are still being discovered [1]. DHPs (also known as Hantzsch esters) are synthetic analogues of reduced nicotinamide adenine dinucleotides (NADHs)—cell “powerhouses” which are coenzymes central to all forms of life (Scheme 1) [2].

**Scheme 2:**
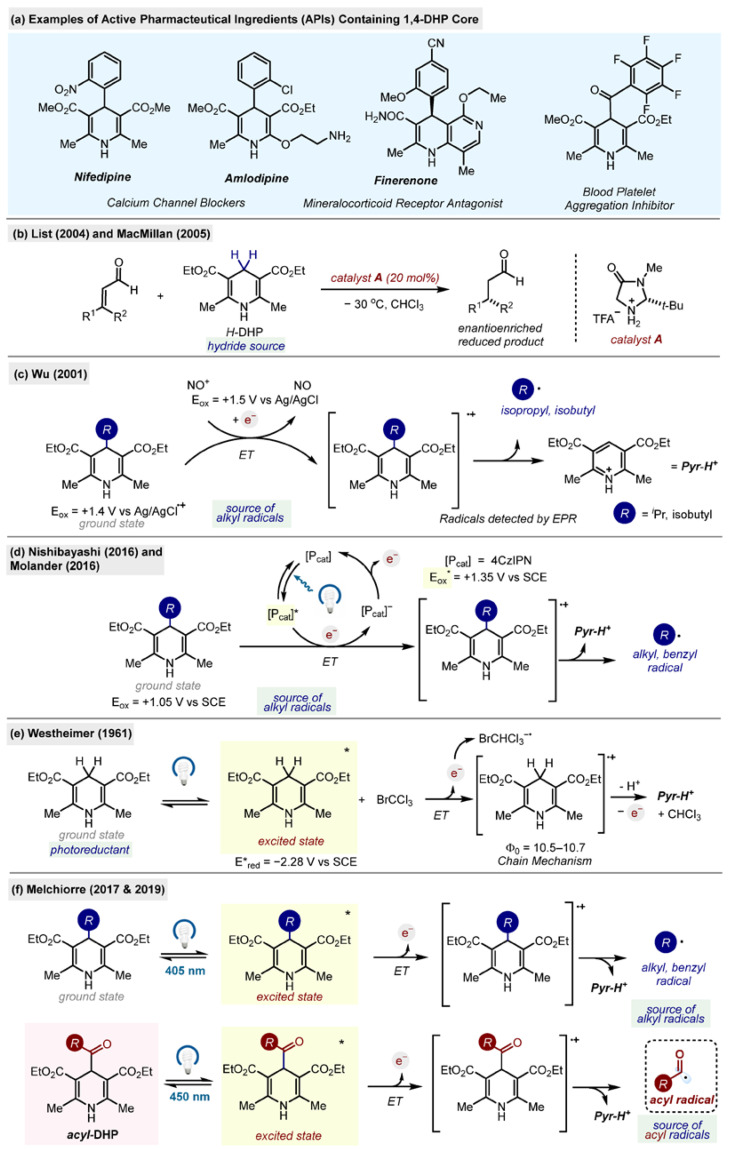
(**a**) Examples of APIs possessing a 1,4-DHP core. (**b**–**f**) Selected developments of 1,4-DHP chemistry: 1,4-DHP as a hydride (**b**), a photoreductant (**e**) and a radical source (**c**,**d**,**f**) [8,9,10,11,12,13,14,15].

Various aspects of DHP chemistry have been reviewed over the years [3,4,5,6]. It has been found that some 1,4-DHP derivatives act as L-type calcium channel blockers, used for the treatment of hypertension. Others have antibacterial, antifungal and anti-inflammatory activity, amongst other potential uses (Scheme 2a) [7].

In the field of organic synthesis, 1,4-DHPs have earned a prominent position as reducing agents and hydride sources [16,17]. It was found that they can reduce conjugated olefins, carbonyls and imines to their corresponding saturated forms [18]. The first examples of asymmetric organocatalytic reactions reported by List and MacMillan, which won the Nobel prize in Chemistry in 2021, featured diethyl 2,6-dimethyl-1,4-dihydropyridine-3,5-dicarboxylate (*H*-DHP) as a hydride source for the enantioselective reduction of iminium ions (Scheme 2b) [8,9]. 

The chemistry of 1,4-DHPs is not limited to their role as a reducing agent and hydride source. In 2001, the group of Wu found that some alkyl-substituted 1,4-DHPs can release alkyl radicals when reacted with strong oxidants such as nitrosonium tetrafluoroborate (NO^+^BF_3_^−^) (Scheme 2c). Isopropyl and isobutyl radicals were detected by EPR [10].

It took more than a decade to translate this mode of reactivity into a synthetic method. In 2016, the research groups of Nishibayashi and Molander reported photocatalyzed alkylations of arenes [11,12]. In both cases, the excited state of the photocatalyst (PC) acts as a strong oxidant which extracts an electron from 1,4-DHP, leading to the formation of a radical cation RC, which then releases an alkyl radical and a pyridinium by-product (Scheme 2d). This development paved the way for the application of 1,4-DHPs as radical precursors, leading to numerous applications in organic synthesis, with thermal and photochemical activation of its ground state [19].

The excited state chemistry of 1,4-DHP was originally investigated by Westheimer, who found that upon excitation, *H*-DHP can act as a strong reducing agent (E*_red_ = −2.28 V vs. SCE), capable of reducing bromotrichloromethane in a chain process (Scheme 2e) [13]. Another breakthrough came from Melchiorre’s group, which observed the formation of alkyl radicals upon direct photoexcitation of alkyl-1,4-DHPs, without the use of external oxidants or photocatalysts with visible light (405 nm). The radicals formed in this process were trapped by nickel salt in a C(sp^2^)–C(sp^3^) cross-coupling reaction (Scheme 2f top) [14]. These alkyl-1,4-DHPs have been extensively exploited in synthesis, quickly gaining prominence as a convenient source of alkyl radicals [19,20,21]. In contrast, generation of acyl radicals from DHP has remained elusive. 

In 2019, Melchiorre’s group found that the neat condensation reaction of glyoxals, ethyl acetoacetate and ethyl 3-aminocrotonate leads to the formation of acyl-1,4-DHPs, which are the topic of this review [15]. Acyl-1,4-DHPs, similar to their alkyl counterparts, can release acyl radicals upon direct excitation with a visible light (Scheme 2f, bottom). Prior to this report, there was only a single example of an acyl-1,4-DHP in the literature, benzoyl-1,4-DHP **1**, with no synthesis provided [22]. Since then, acyl-1,4-DHPs have been used in synthesis in numerous applications, as reductants and acyl radical precursors, and they have earned their position as a convenient source of acyl radicals for organic synthesis. 

This review article discusses the applications of acyl-1,4-DHPs in organic synthesis since their introduction in 2019. Its objective is to present a coherent summary of the synthetic scope of these reagents to date, along with the modes of activation and mechanisms of action. Carbamoyl radicals, which are generated in a similar way, are outside of the scope of this review [23].

### 1.1. Synthesis

**Scheme 3:**
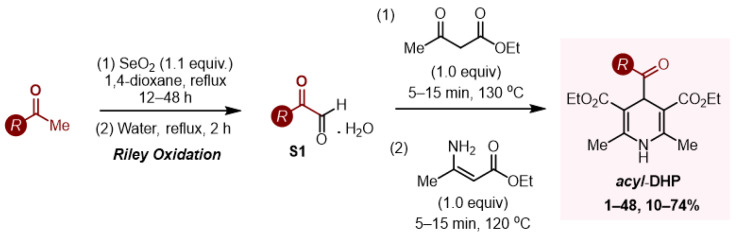
Preparation of acyl-1,4-DHPs from methyl ketones.

Acyl-1,4-DHPs can be readily prepared from corresponding glyoxals or glyoxal hydrates in a single step in multigram quantities by a modified Hantzsch procedure (Scheme 3) [15]. Many glyoxals or glyoxal hydrates (**S1**) are commercially available, although relatively expensive. Phenylglyoxal hydrate, for example, costs €74.3 for 5 g (Sigma-Aldrich, St. Louis, MO, USA, June 2024). Alternatively, glyoxals can be prepared via Riley oxidation of methyl ketones in an operationally simple procedure, involving refluxing of methyl ketones with SeO_2_ in 1,4-dioxane. In most cases, the reaction is clean and the product can be purified by recrystallization [15].

Glyoxals or glyoxal hydrates **S1** can be reacted with ethyl acetoacetate at 120 °C in a Knoevenagel condensation, followed by a cyclisation with ethyl 3-aminocrotonate to produce acyl-1,4-DHPs **1–48** in moderate to high yields. 

**Figure 1:**
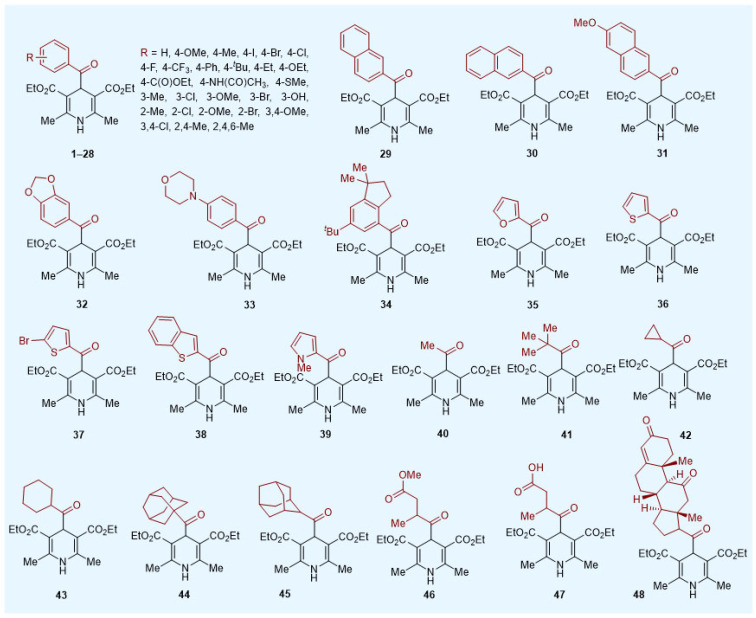
Reported acyl-1,4-DHP radical precursors **1–48**.

Products can be purified by column chromatography and/or by recrystallization in good to moderate yields. So far, 48 different acyl-DHPs have been prepared this way (Figure 1), containing a diverse set of benzoyl **1–34**, heterobenzoyl **35–39** and alkyloyl **40–48** groups.

In most cases, acyl-DHPs are crystalline, bench-stable solids, which can be stored at room temperature for a long period without the risk of decomposition or the necessity of excluding light, air or moisture. These properties make them desirable and convenient reagents for the controlled release of acyl radicals [24].

### 1.2. Reactivity and Mechanism of Action 

**Scheme 4:**
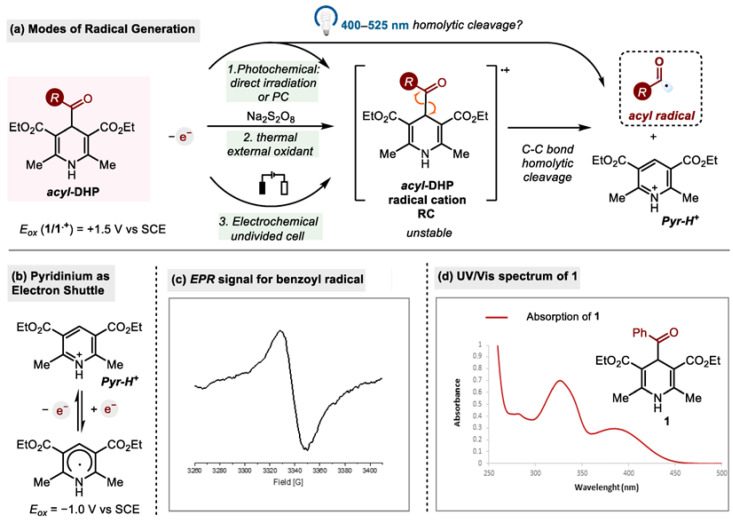
Mechanism of action of acyl-1,4-DHP. (**a**) Three modes of radical generation: photochemical, chemical (thermal) and electrochemical and proposed mechanism. (**b**) Pyridinium salt by-product acting as an electron shuttle. (**c**,**d**) EPR signal for benzoyl radical and UV-Vis spectrum of **1** (reproduced from [15]).

Acyl-1,4-DHPs release acyl radicals upon direct excitation with visible light (390–525 nm)/direct sunlight, electrochemically [25] or chemically (thermally), when in contact with strong oxidants (Scheme 4a) [26]. Spectroscopic analysis of **1** reveals an absorbance peak tailing towards the visible range, with a peak of 390 nm (Scheme 4c). It has been found that radicals are produced when **1** is irradiated with a green LED (525 nm); however, this process is sluggish and takes multiple hours for its completion. Evidence of radical generation was provided by EPR (Scheme 4d) and TEMPO trapping experiments [15]. 

The exact mechanism of the radical formation is not immediately obvious. It was originally proposed that the DHP acts as a photoreductant (estimated *E_ox_* (1*/1^.+^) = −1.10 V vs. SCE), capable of transferring an electron from its excited state to a pyridinium salt by-product **Pyr-H^+^** (*E_ox_* (**Pyr-H^+^/PyrH^.^**) = −1.0 V vs. SCE) [27] or to oxygen in air [28] to form an unstable radical cation RC and stabilized reduced pyridinium radical **Pyr-H^.^**, which acts as an electron shuttle (Scheme 4b). Unstable RC immediately decomposes, forming a **Pyr-H^+^** and an acyl radical. Acyl-1,4-DHPs can also act as single-electron reductants in their ground state (*E_ox_* (**1/1^.+^**) = +1.5 V vs. SCE), undergoing reactions with strong oxidants such as Na_2_S_2_O_8_ to form unstable RC, which releases acyl radicals via C-C homolytic cleavage [24]. It was also found that the same RC intermediate can be accessed by electrochemical oxidation [25,29].

The reduced form of Hantzsch pyridine, **Pyr-H**, is a potent single-electron reducing agent capable of delivering an electron to products of a reaction between an acyl radical and radical acceptors to form closed-shell products. This hypothesis is supported by computational studies of reaction intermediates performed by the Melchiorre group [27]. This is also corroborated by DFT studies of the properties of acyl-1,4-DHP radical cations by Yan et al., where it was found that **1^+.^** is an excellent radical donor with Δ*G* ^o^_RD_(XRH˙^+^) = −3.13 kcalmol^−1^, Δ*G* ^≠^_RD_(XRH˙^+^) = 3.44 kcalmol^−1^ and k_2_ = 1.85 × 10^10^ M^−1^s^−1^ for the formation of acyl radicals [30,31]. The stepwise mechanistic proposal, where an electron is transferred prior to the radical release, is also confirmed by other experimental studies which are discussed in this review article. An alternative hypothesis, whereby the C-C bond undergoes direct homolytic cleavage upon excitation, was also suggested and supported by computational studies, indicating elongation of the C-C σ-bond upon excitation (vide infra) [32].

In summary, acyl-1,4-DHPs play a dual role as single-electron reductants (in ground and excited state) and sources of acyl radicals, being able to deliver an acyl radical and a single electron in the same chemical system. 

## 2. Reactions of Acyl-1,4-DHPs

Acyl-1,4-DHPs were found to be useful and convenient reagents in organic synthesis. They have been used extensively as radical sources in asymmetric radical conjugate additions and in oxidative and reductive functionalization of *N*-heterocycles, in addition to activated and non-activated C-C double and triple bonds. They have been found to form charge transfer complexes (electron donor–acceptor, EDA) with electron-deficient species, leading to unusual patterns of reactivity, and they have been applied to radical–polar cross-over reactions for the synthesis of cyclopropanes. Acyl radicals, generated from acyl-1,4-DHPs, can also successfully undergo trifluoromethylthiolation, trifluoromethylselenolation and hydroacylation of azodicarboxylic acid derivatives, amongst other functions. 

### 2.1. Enantioselective Radical Conjugate Additions

**Scheme 5:**
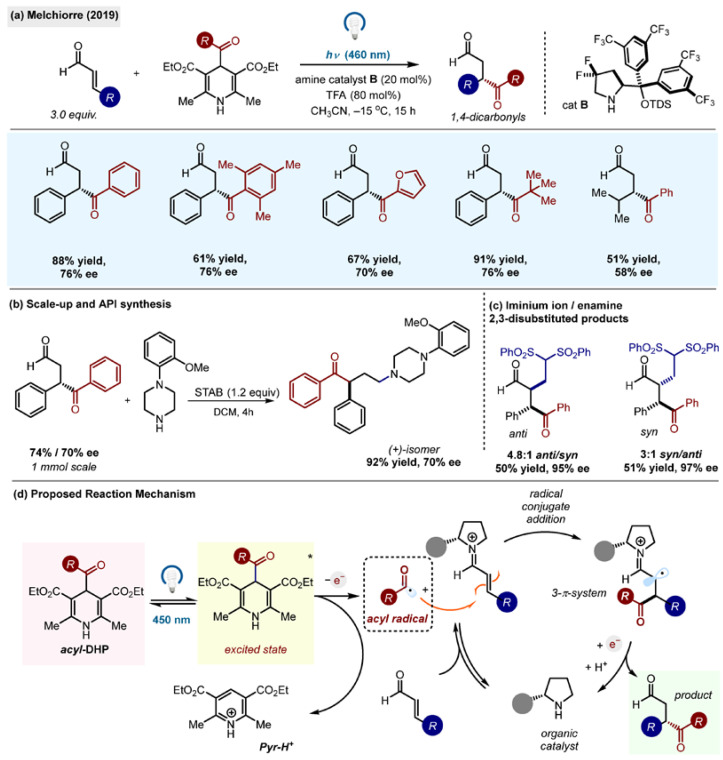
Enantioselective radical conjugate additions utilizing acyl-1,4-DHPs. (**a**) Asymmetric addition of acyl radicals to enals (Melchiorre 2019) [15]. (**b**) Scale-up and synthesis of serotonin 5HT_1A_ receptor antagonist. (**c**) 2,3-Disubstituted products of one pot iminium ion/enamine catalysis. (**d**) Proposed reaction mechanism.

The first application of acyl-1,4-DHPs in organic synthesis was reported by the Melchiorre group, which used them for asymmetric conjugate radical additions of acyl radicals to iminium ions to produce enantioenriched 1,4-dicarbonyl compounds in high yields and ee’s (Scheme 5a) [15]. Enantioselective radical reactions are generally rare and notoriously difficult due to the high reactivity of radical species [33]. In this work, imine organocatalysis was found to be compatible with the DHP radical system. The organic catalyst used, **B**, was the modification of Jorgensen’s catalyst derived from proline.

Acyl radicals were generated by direct irradiation of acyl-1,4-DHPs without the necessity of an external oxidant or a photocatalyst/photosensitizer. Melchiorre’s group reported the first synthetic protocol for this class of reagents, preparing 13 examples of 1,4-acyl-DHPs (aryl, heteroaryl and alkyl R groups, Scheme 5a). The practical utility of the method was demonstrated by the scale up and enantioselective synthesis of serotonin 5HT_1A_ receptor antagonist (Scheme 5b). 2,3-Difunctionalized 1,4-dicarbonyl compounds were also prepared in one pot cycle-specific iminium/enamine catalysis (Scheme 5c). This report marks the first asymmetric capture of acyl radicals. The mechanism (Scheme 5d) involves conjugate radical addition of acyl radicals to iminium ion intermediates, followed by the single-electron reduction and acid-catalyzed cleavage of the amine organocatalyst.

**Scheme 6:**
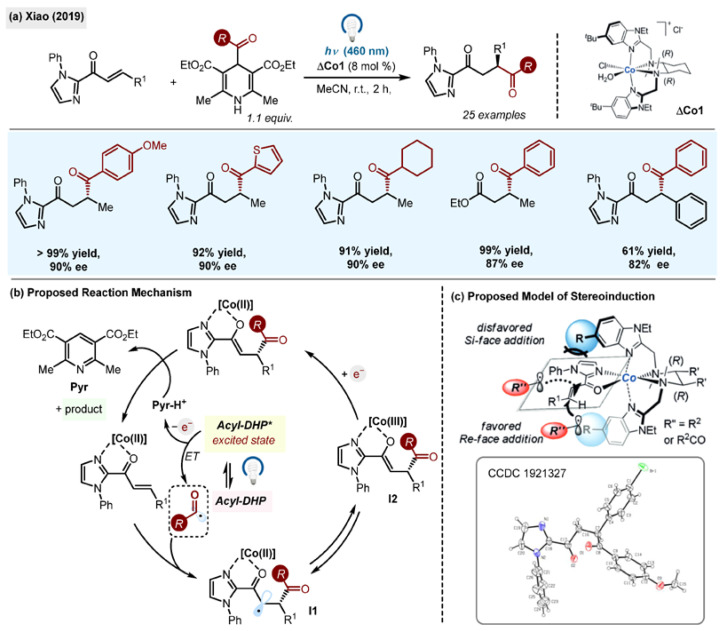
(**a**) Asymmetric addition of acyl radical to enones (Xiao 2019) [34] and selected examples. (**b**) Proposed reaction mechanism. (**c**) Proposed model of stereoinduction (reproduced from [34]).

The group of Xiao reported visible light-triggered asymmetric radical conjugate addition of 1,4-DHP-derived alkyl and acyl radicals to enones possessing a directing group (1-phenyl-1H-imidazole = DG) utilizing the rationally designed octahedral chiral-at-metal cobalt catalyst **ΔCo1** (Scheme 6a) [34]. 

The 1,4-dicarbonyl products were prepared with an excellent degree of stereoselectivity and in high yields from acyl-1,4-DHPs (Scheme 6a). In contrast to alkyl-1,4-DHPs, acyl-1,4-DHPs did not require a photocatalyst for their activation since the radicals were generated through direct irradiation.

In addition to imidazole DG containing substrates, it was possible to achieve reactivity and enantioselectivity for an ethyl ester instead of a DG in one instance. The proposed mechanism (Scheme 6b) involves the direct excitation of acyl-1,4-DHPs, which release acyl radicals upon electron transfer (ET). The acyl radical adds to the β-position of the **ΔCo1**-activated enone and the resulting radical intermediates **I1** or **I2** are reduced by either **Pyr-H^.^** or DHP* (Scheme 6c).

The quantum yield measured for this reaction was found to *ϕ* = 0.861, which suggests a direct excitation pathway rather than a radical chain mechanism. A model for the stereoinduction was proposed, favouring radical conjugates’ addition to the *Re*-face of an enone due to the steric interactions with a *^t^*Bu group of the ligand (Scheme 6c). 

### 2.2. Reductive C-H Hydoxyalkylation of Quinolines and Isoquinolines

**Scheme 7:**
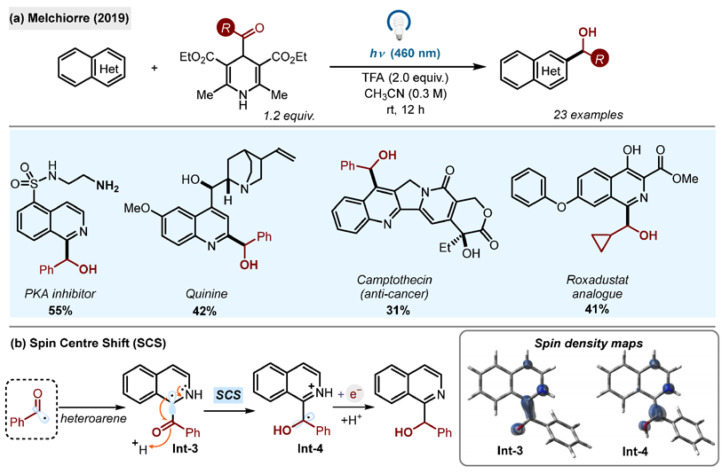
(**a**) Reductive C-H hydroxyalkylation of quinolones and isoquinolines and selected examples of late-stage functionalization of pharmaceutical agents [27]. (**b**) Proposed spin-centre shift (SCS) and calculated spin density maps (reproduced from [27]).

The dual function of 1,4-acyl-DHP as a reductant and an acyl radical source was exploited by the Melchiorre group for the C-H hydroxyalkylation of quinolines and isoquinolines (Scheme 7a) [27]. The addition of acyl radicals to these heterocycles formed unexpected, reduced alcohol products in place of the expected Minisci adducts—ketones.

This was due to the lack of external oxidants in the reaction mixture. The method was applied to late-stage C-H hydroxyalkylation of APIs under mild conditions (Scheme 7a), with a remarkable functional group tolerance (e.g., primary amines and alcohols). 

Computational studies of the reaction mechanism revealed a new type of spin-centre shift (SCS), where the lone pair of electrons acts as a leaving group in an unusual 1,2-radical migration (Scheme 7b).

It was also proposed that the pyridinium salt, **Pyr-H^+^**, can act as an internal oxidant to initiate the ET process from DHP* and the **Pyr-H^+^/PyrH^.^** couple can act as an electron shuttle. These findings were supported by electrochemical measurements and deuterium labelling studies. 

### 2.3. Oxidative Acylation of N-Heteroarenes

Many classes of *N*-heteroarenes, being secondary metabolites, are pharmacophores exhibiting important medicinal properties. Oxidative radical addition to *N*-heteroarenes, also known as the Minisci reaction [35,36], has been a playground for radical organic chemistry for many years. In the presence of an oxidant, alkyl or acyl radicals added to the 2- or 4- position of acid-activated *N*-heteroarenes can form alkylated or acylated products. This strategy enables the derivatization of *N*-heteroarenes without the necessity of de novo synthesis, which is highly relevant for drug research. In the examples below, acyl-1,4-DHPs were activated chemically by strong oxidants and electrochemically. 

**Scheme 8:**
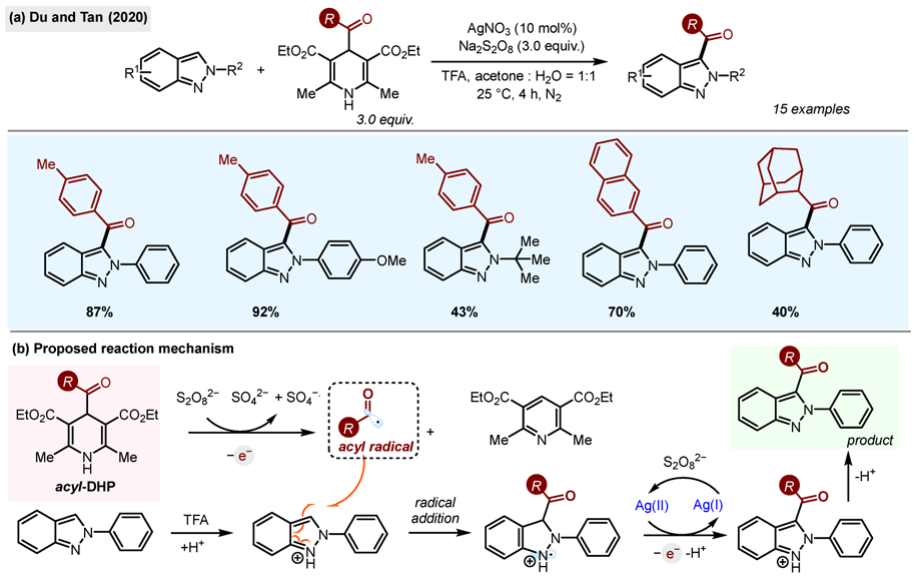
(**a**) Oxidative 3-acylation of *2H*-indazoles by Du and Tang (2020) [26], selected examples. (**b**) Proposed reaction mechanism.

Du and Tan reported the synthesis of 3-acylated *2H*-indazoles (Scheme 8) using acyl-1,4-DHPs and a classic Minisci oxidative system: AgNO_3_/Na_2_S_2_O_8_ [26]. 2*H*-Indazoles are common motifs in bioactive natural products and pharmaceutical agents as bioisosteres of indoles, benzimidazoles and purines. The reaction was successful for 2-aryl- and 2-alkyl-substituted *2H*-indazoles, although alkyl groups gave lower yields. 

The scope of acyl radicals included aryl, heteroaryl and alkyl moieties, including the challenging adamantyl substituent. The reactions were scalable (to 5.15 mmol), selective for the 3-position, and no side products were observed. In order to demonstrate the utility of the method, the authors synthesized an anti-inflammatory agent and NS5B polymerase inhibitor C, shortening the existing synthetic routes to both compounds.

The mechanism for the reaction was probed using TEMPO inhibition and TEMPO adducts were isolated. Based on that finding, the single-electron oxidation of acyl-1,4-DHP by Na_2_S_2_O_8_ was proposed as the initial step of the reaction.

**Scheme 9:**
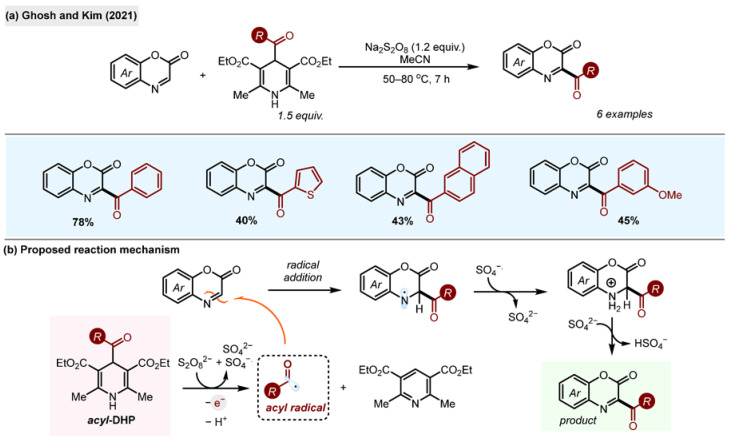
(**a**) Oxidative acylation of 2-benzoxazninones by Ghosh and Kim (2021) [37] and selected examples. (**b**) Proposed reaction mechanism.

Another important class of *N*-heteroarenes acylated with 1,4-acyl-DHPs is 2-benzoxazninones [37]. 2-Benzoxazinones are of interest to medicinal chemists due to their potent bioactivities. Cephaloindole A, for example, exhibits anti-cancer properties. Ghosh and Kim reported a Minisci-type oxidative acylation of 2-benzoxazinones with acyl-1,4-DHPs using the inexpensive Na_2_S_2_O_8_ as an oxidant (Scheme 9a).

The scope of the reaction included substituted starting materials and an aryl, heteroaryl and alkyl acyl radical. The method was scalable to 1 g. The reaction proceeds with a classical Minisci radical pathway (as evidenced by isolating a TEMPO adduct) involving single-electron oxidation of acyl-1,4-DHP by an external oxidant, Na_2_S_2_O_8_ (Scheme 9b). 

**Scheme 10:**
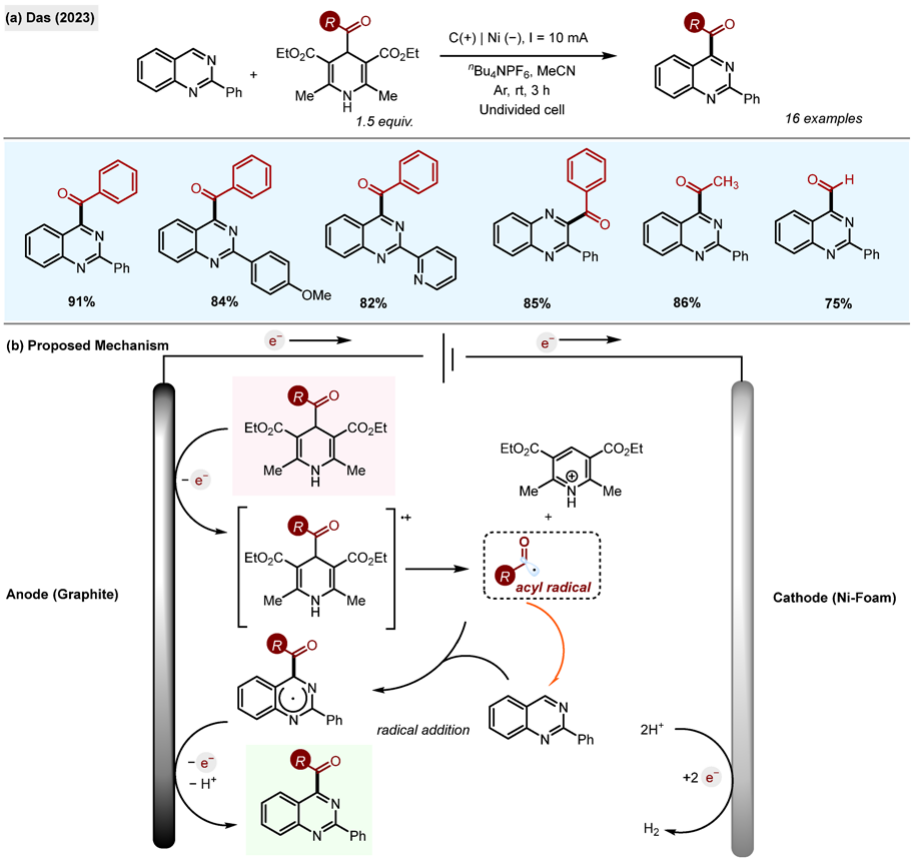
(**a**) Electrochemical acylation of quinazolines and quinoxalines (2023) by Das [29]. (**b**) Proposed mechanism.

Quinazolines and quinoxalines are two other important classes of *H*-heteroarenes with exceptional and diverse medicinal properties, with anti-tumour, anti-inflammatory, antioxidant, antibacterial, anti-viral and anti-hypertensive activities, amongst others [38]. Current synthetic methods of C4 substitution of quinazolines and quinoxalines are underdeveloped from the point of view of sustainable chemistry. In order to address this limitation, the group of Das developed an electrochemical protocol for Minisci-type alkylation and acylation of quinazolines and quinoxalines with 1,4-DHPs (Scheme 10a) [29].

The optimized conditions involved performing the reaction in an undivided electrochemical cell with graphite (anode) and nickel (cathode) electrodes, under constant current (10 mA) with CH_3_CN/trifluoroethanol (TFE, 3:1) as a solvent and *^n^*Bu_4_NPF_6_ as an electrolyte. 

The reaction was successful for benzoyl, acetyl and formyl acyl-1,4-DHP, producing acylated quinazolines and quinoxalines in high yields. Electrochemical studies of **1** confirmed the easy oxidation of **1** to **1^.+^** at the anode, generating acyl radicals. The authors found that addition of trifluoroethanol (TFE) to CH_3_CN and its use as a co-solvent resulted in a decrease in E_ox_ (**1/1^.+^**) from +1.9 V vs. SCE to +1.5 V vs. SCE, likely due to the chemical interactions between TFE and DHP. This observation can explain the increase in the reaction yield in this solvent mixture compared to CH_3_CN on its own. The proposed mechanism of radical generation involves single-electron oxidation of acyl-1,4-DHP.

**Scheme 11:**
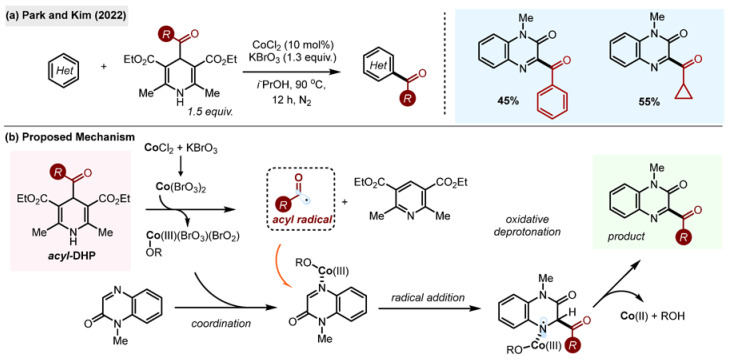
(**a**) Oxidative acylation of *N*-heteroarenes using KBrO_3_/Co(II) couple by Park and Kim (2022) [39]. (**b**) Proposed mechanism.

Another method of chemical activation of 1,4-DHPs was discovered by Park and Kim, who used a KBrO_3_/CoCl_2_ couple for single-electron oxidation of these radical precursors (Scheme 11a) [39]. A diverse set of *N*-heteroarenes were functionalized this way, including azauracils, quinoxalinones, pyrazinones, pyridones, quinolones, quinazolinones, xanthines, chromones and others. Although the protocol is mostly focused on the use of alkyl-DHPs, the authors reported two examples of successful acylation, using acyl-1,4-DHPs—benzyoyl and alkyloyl. It is proposed that the active species in this reaction might be Co(BrO_3_)_2_, which oxidizes 1,4-DHPs to form radical cations (Scheme 11b). 

### 2.4. Radical Addition to C-C Double Bonds

Addition of acyl radicals across unsaturated C-C double bonds is a convenient way of installing a keto group while forming a new C-C bond. The vast majority of synthetic strategies require the use of activated alkenes (possessing an electron-withdrawing group-EWG), as coupling partners, while the application of unactivated olefins is much less common. Two enantioselective examples of the addition of acyl radicals generated from acyl-1,4-DHP to activated olefins were discussed above (Scheme 5a) [15,33]. 

**Scheme 12:**
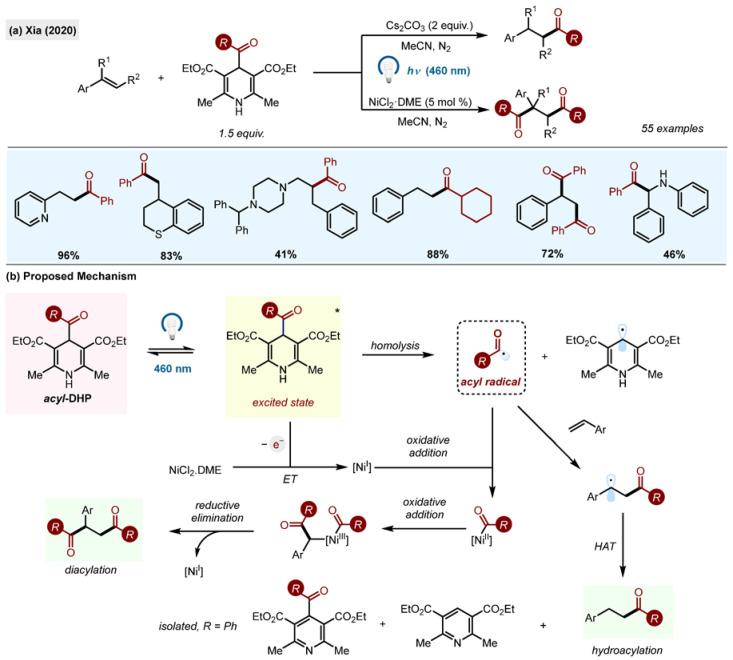
(**a**) Radical addition to C-C double bonds: hydroacylation and dihydroacylation of styrenes by Xia (2020) [40]. (**b**) Proposed mechanism.

Following these reports, the group of Xia disclosed hydroacylation and dihydroacylation of styrenes through direct excitation of acyl-1,4-DHPs with visible light (Scheme 12) [40]. The reaction tolerated multiple functional groups, including tertiary amines. The authors disclosed an extensive reaction scope of styrene acceptors, utilizing aryl, heteroaryl and alkyl acyl radicals. Interestingly, acyl radicals were also added to aldimines, although with lower yields. 

The addition of two equivalents of a base (Cs_2_CO_3_), which increased the yield for the model reaction from 26% to 87%, was critical for the efficiency of the reaction. It was also found that the presence of air caused a significant decrease in the reaction yield, likely due to oxygen quenching of acyl radicals. The addition of 5% of NiCl_2_.DME allowed the synthesis of 1,4-dicarbonyl compounds by double hydroacylation. The benzyl radical, formed after the acyl radical addition to a styrene, was trapped by Ni, followed by the addition of an acyl radical to a metal centre and reductive elimination of the diacylated product. 

The method was scaled up to 15 mmol and applied to the late-stage functionalization of structurally complex molecules. The radical mechanism was confirmed by the isolation of TEMPO adducts. The authors proposed homolysis of the excited state of DHP* as the mechanism for radical generation.

**Scheme 13:**
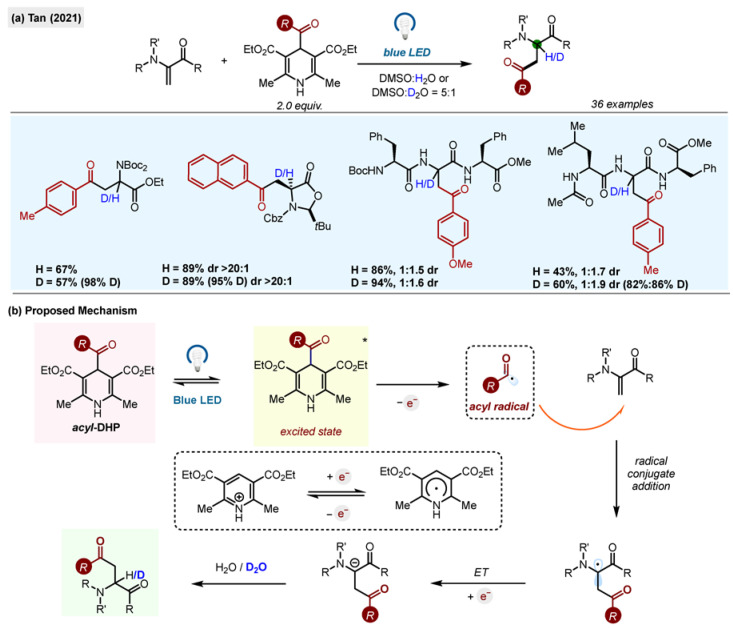
(**a**) Hydroacylation of peptides by Tan (2021) [41]. (**b**) Proposed mechanism.

The dual role of 1,4-acyl-DHP as a single-electron reductant and acyl radical precursor was exploited by Tan et al., who reported a synthetic protocol for the preparation of unnatural β-acylated α-amino acids by photochemical addition of acyl radicals to dehydroalanines, which are also activated olefins (Scheme 13) [41]. The intermediate anion, produced after the reduction of the adduct formed by acyl radical addition to a C-C double bond, was quenched with D_2_O, allowing for the selective introduction of deuterium atoms to the α-position of synthesized amino acids (96% deuterium incorporation for the model reaction). When chiral Karady–Beckwith alkenes were used as substrates, the deuteration of the anion occurred with a high degree of diastereoselectivity (20:1). 

The process exhibited remarkable scope both for the radical donor and the olefin acceptor, enabling functionalization of dehydroalanine derivatives, di- and tri- peptides (e.g., Ac-Leu-Ser-Phe-OMe). Various protecting groups for N and C were tolerated, including Boc and phthalimide. 

Mechanistic investigations confirmed a radical mechanism by detecting TEMPO adducts by MS. The possibility of an electron donor–acceptor (EDA) complex was excluded by spectroscopic studies.

**Scheme 14:**
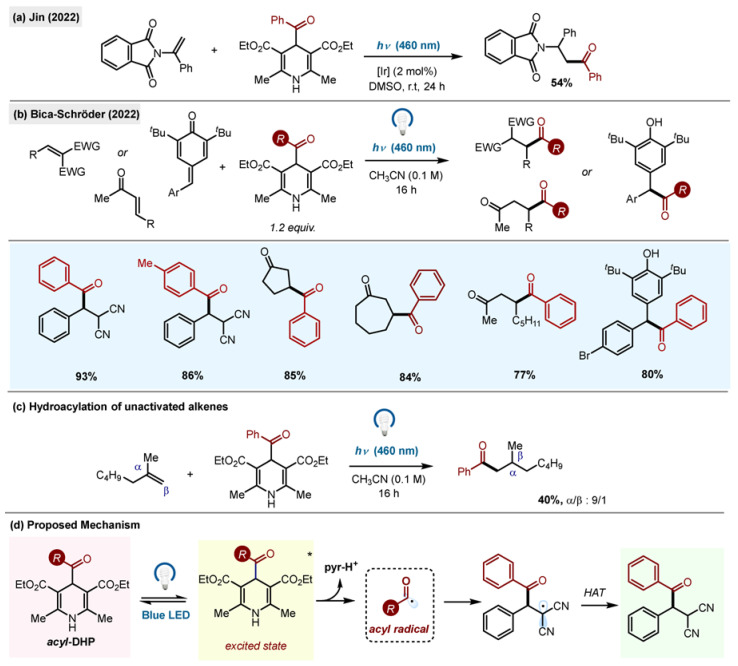
(**a**) Hydroacylation of styrene by Jin (2022) [42]. (**b**) Radical additions to activated and unactivated olefins by Bica-Schröder (2022) [43]. (**c**) Hydroacylation of unactivated alkenes by Bica-Schröder. (**d**) Proposed mechanism.

The authors proposed the ET from DHP* as the initial step in the reaction sequence and following the previous hypothesis from the Melchiorre group [27] indicated that **Pyr-H^+^/PyrH^.^** couple was involved in a process, and it facilitated the reduction of the final radical into an anion (Scheme 14b). This report marks the first photochemical acylation of polypeptides.

The single example of hydroacylation of styrene was reported by Jin (Scheme 14a) [42]. Extensive study of the scope of reaction of acyl radicals derived from acyl-1,4-DHPs with C-C double bonds was performed by the group of Bica-Schröder (Scheme 14b,c) [43]. The authors reported efficient acyl transfer to activated alkenes and *p*-quinone methides. Importantly, it was found that the photogenerated acyl radicals could undergo additions to unactivated olefins with moderate efficiency. The addition products were isolated in 32–48% yields.

**Scheme 15:**
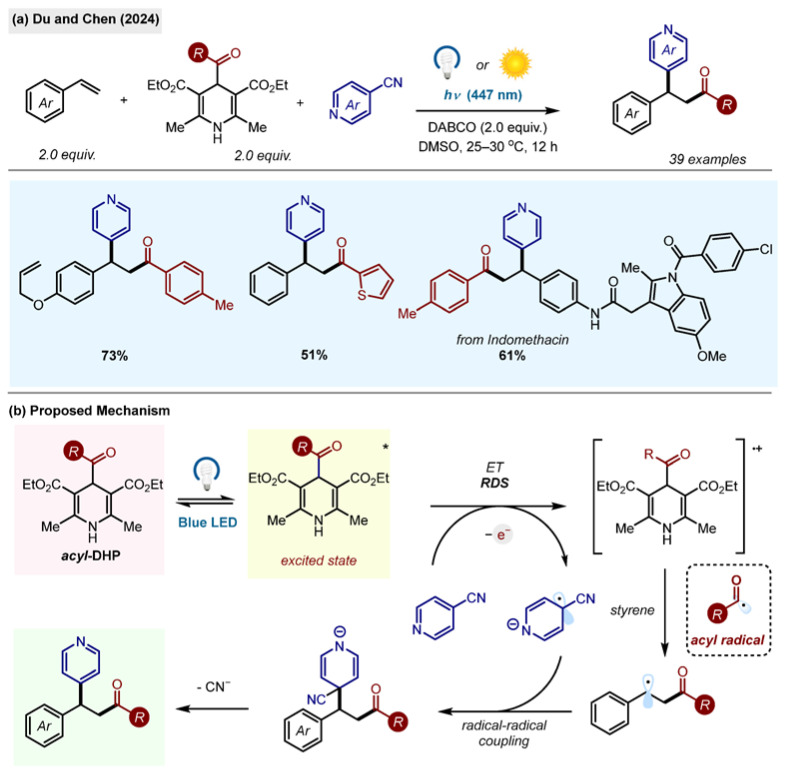
(**a**) Acylative pyridylation of styrenes by Du and Chan (2024) [44]. (**b**) Proposed mechanism.

Du and Chen set out to use styrene, 4-cyanopyridine and acyl-1,4-DHPs in the visible light-induced photocatalyst-free synthesis of *β*-aryl-*β*-pyridinyl ketones (Scheme 15) [44]. The selection of solvent greatly affected the yield, with DMSO resulting in the highest efficiency. DABCO was used as a base to neutralize the HCN formed during the reaction. The generality of the reaction was investigated with styrene with varying electronic substituents, of which electron-withdrawing groups resulted in the greatest yields. 

The proposed mechanism starts with the excitation of the acyl-1,4-DHP, which then triggers a single-electron reduction of the cyanopyridine forming the acyl radical cation and cyanopyridine radical anion. Fragmentation of the radical cation then takes place, liberating the acyl radical which reacts with styrene. The resulting benzyl radical couples with the cyanopyridine radical anion, resulting in the formation of the final desired β-aryl-β-pyridinyl ketone. The reaction was also found to proceed under direct sunlight, but with a lower yield. 

### 2.5. Radical Addition to C-C Triple Bonds

**Scheme 16:**
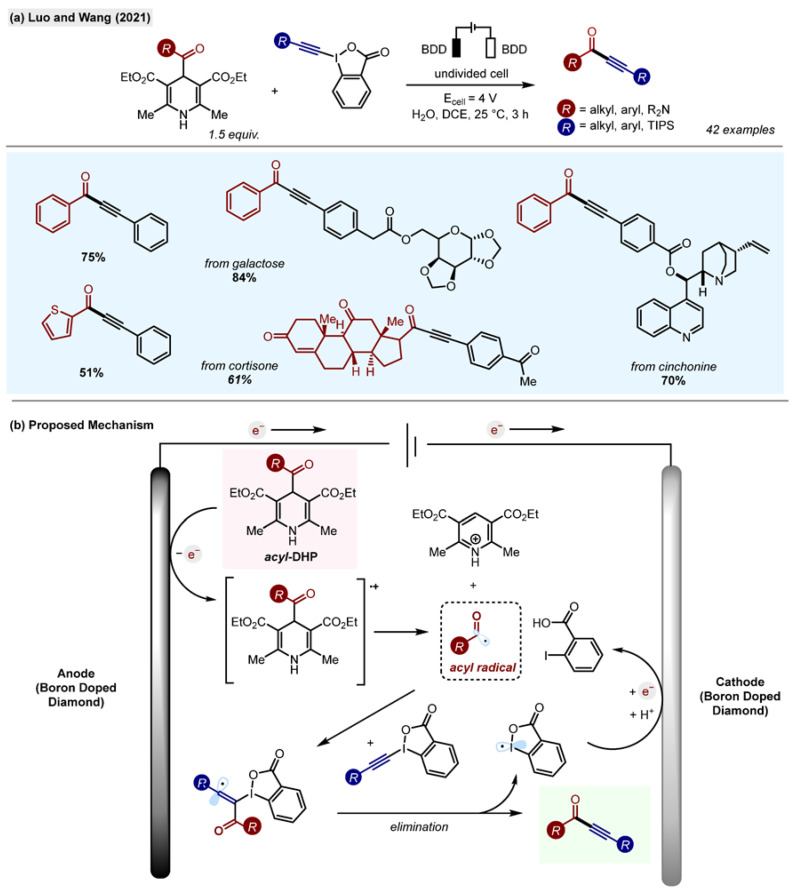
(**a**) Electrochemical radical addition to C-C triple bonds by Luo and Wang (2021) [25]. (**b**) Proposed mechanism.

A single report describing additions of 1,4-DHP-derived acyl radicals to alkynes was disclosed by Luo and Wang (Scheme 16) [25]. Acyl-1,4-DHPs were electrochemically activated by an anodic ET and the generated acyl radicals reacted with hypervalent iodine (III) to form ynones and ynamides in high yields. 

The reactions were carried out in an undivided cell under constant voltage (4.0 V) with boron-doped diamond (BDD) used as a cathode and anode and *n*-Bu_4_NPF_6_ in dichloroethane as an electrolyte in the presence of water (10 equiv.). Ynones with a variety of functional groups, such as esters, alkyl and aryl halides, amines, *N*-heterocycles and others were prepared. The scope of the acyl-1,4-DHP employed was also broad and it included aryl, heteroaryl and alkyl substituents. However, reactions featuring the α- *^t^*Bu group were unsuccessful.

In order to demonstrate the utility of the method, the authors carried out the late-stage functionalization of complex pharmaceutical molecules, including steroids and sugars (Scheme 16). Electrochemical studies confirmed that the ET from acyl-1,4-DHP to the anode was necessary for its activation.

### 2.6. Acylation of Imines

Nucleophilic addition to imines is an attractive way of synthesizing amines via the formation of a new C-N bond [45,46]. While polar nucleophilic additions to imines have been widely explored, including enantioselective variants often leading to chiral amines with a high degree of stereoselectivity, radical 1,2 additions to imines are much less common [47].

**Scheme 17:**
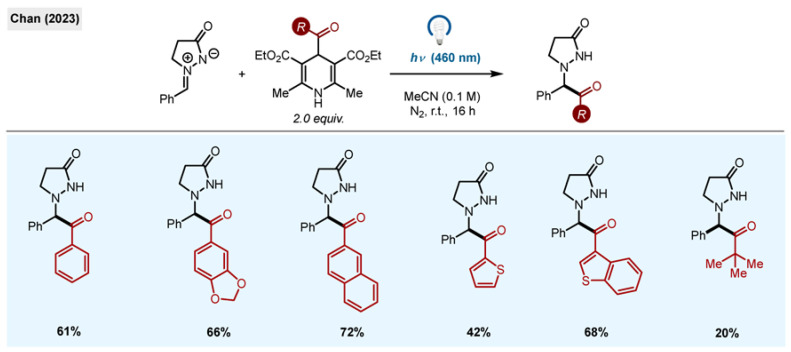
Acylation of imines: addition of acyl radicals to azomethine imines by Chan (2023) [48].

The group of Chan found that acyl radicals, generated from 1,4-acyl-DHP, react efficiently with azomethine imines to form 1-acyl 3-pyrazolidinones in high yields (Scheme 17) [48]. 3-Pyrazolidinones are important *N,N*-heterocyclic compounds, acting as building blocks in synthesis and exhibiting biological activities. It was found that the reaction proceeded upon direct irradiation of acyl-1,4-DHPs with visible light, and, in contrast to alkyl-1,4-DHPs, did not require a photocatalyst. The authors reported acyl transfer from aryl, heteroaryl and alkyl acyl-1,4-DHPs, although the yields for the *tert*-butyl substituent were significantly lower (20%) compared with benzoyl (62%). 

Enantioselective addition of acyl radicals to azaarene-substituted ketimines was achieved by Jiang using acyl-1,4-DHPs under photoredox conditions using chiral Brønsted acid catalysis (Scheme 11b) [49]. The products of the reaction, azaarene-substituted tertiary amines, are known to exhibit pharmaceutical activities and they are notoriously difficult to prepare with a high degree of stereoselectivity. Here, the advantage of using a radical methodology lies in overcoming the problem of the *Z/E* configuration of ketimines since the radical anion intermediates for *Z* and *E* forms possess the same configuration. 

**Scheme 18:**
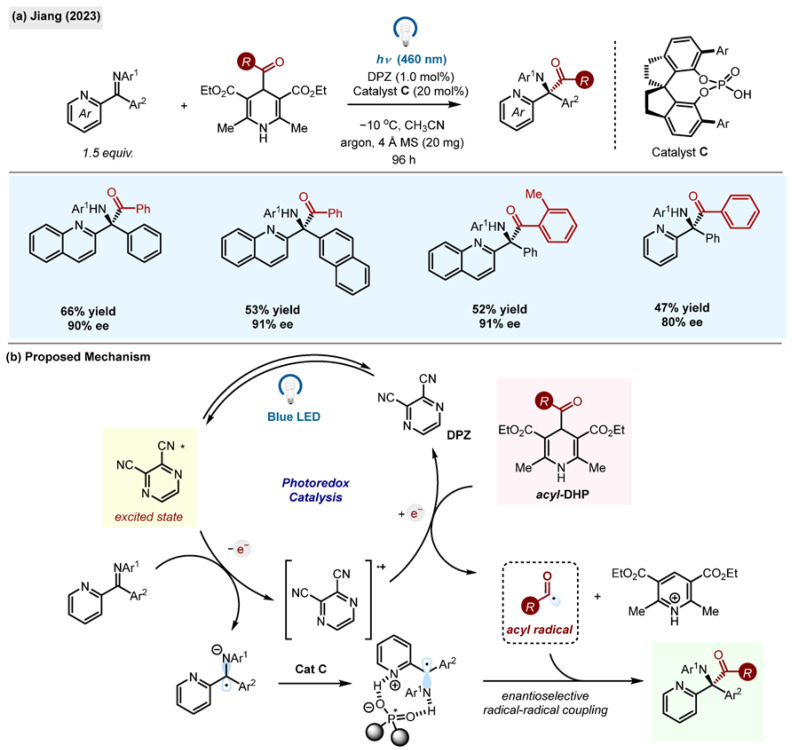
(**a**) Asymmetric formation of tertiary amines by acyl radical addition to azaarene substituted ketimines by Jiang (2023) [49]. (**b**) Proposed mechanism.

The reaction (Scheme 18) relied on a dual catalytic system, exploiting a dicyanopyrazine photocatalyst (DPZ) and a chiral phosphoric acid (**Cat C**). Under blue light irradiation, at −10 °C, in the presence of molecular sieves, acylated tertiary amines were formed from benzoyl acyl-1,4-DHP with high yields and excellent stereocontrol.

The scope of the reaction included quinolines and pyridines as azaarene substituents. Importantly, the reaction utilized *Z*/*E* mixtures of ketimines, with the *Z*/*E* ratio having no impact on the configuration of the final products. A chiral H-bonding phosphoric acid catalyst (**Cat C**) can coordinate both nitrogen atoms of azaarene imines, providing stereochemical control of a radical coupling step. This remarkable achievement was accomplished despite the existence of the background racemic reaction.

Two plausible mechanisms for this transformation were proposed. Direct photoexcitation of ketimines may trigger a single-electron oxidation of 1,4-DHPs, generating a radical anion, a radical and pyridinium. The product is formed by enantioselective radical–radical coupling. Alternatively, the reduction of ketimines may be performed by the excited state of a photocatalyst DPZ*, which is regenerated by single-electron oxidation of the DHP. Both radical species combine to form enantioenriched products (Scheme 18b).

**Scheme 19:**
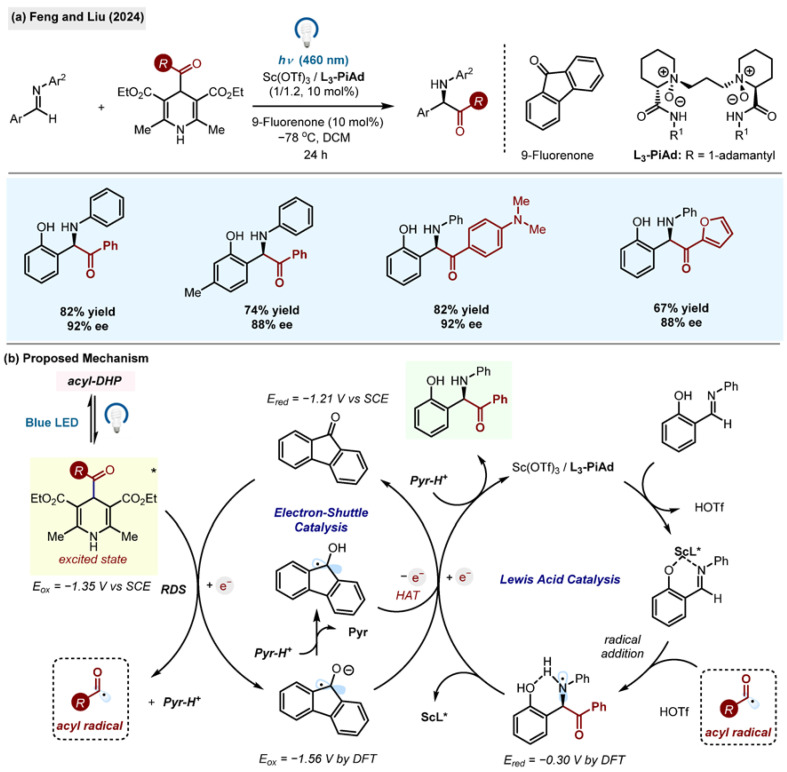
(**a**) Asymmetric acylation of aldimines by Feng and Liu (2024) [50]. (**b**) Proposed mechanism.

Recently, the research groups of Feng and Liu reported that visible light promoted enantioselective acylation of aldimines with acyl-1,4-DHP as an acyl radical precursor (Scheme 19a) [50]. In the presence of a Lewis acid–Sc(OTf)_3,_ a chiral ligand **L_3_-PiAd** and mediated by 9-fluorenone (FLN) electron-shuttle catalysis, DHP-derived acyl radicals react with aldimines to yield α-tertiary amino ketones with high yields and enantioselectivities. The acyl radical is generated by direct excitation of acyl-1,4-DHP with visible light, followed by electron transfer from the excited-state DHP (−1.35 V vs. SCE) to the FLN (−1.21 V vs. SCE) electron acceptor. This step was also found to be a rate-determining step for the whole process (Scheme 19b). The electron transfer from DHP* to FLN was evidenced by the Stern–Volmer quenching experiment.

### 2.7. Hydroacylation of Azodicarboxylic Acid Derivatives

Acyl hydrazides are an important class of compounds that possess a variety of biological activities. In the past, acyl hydrazides have been synthesized by employing transition-metal-based photocatalysts or organic dyes. The work put forward by Tan et al. displays a novel methodology towards the conjugate addition of acyl-1,4-DHP-derived acyl radicals to azo-compounds [51].

**Scheme 20:**
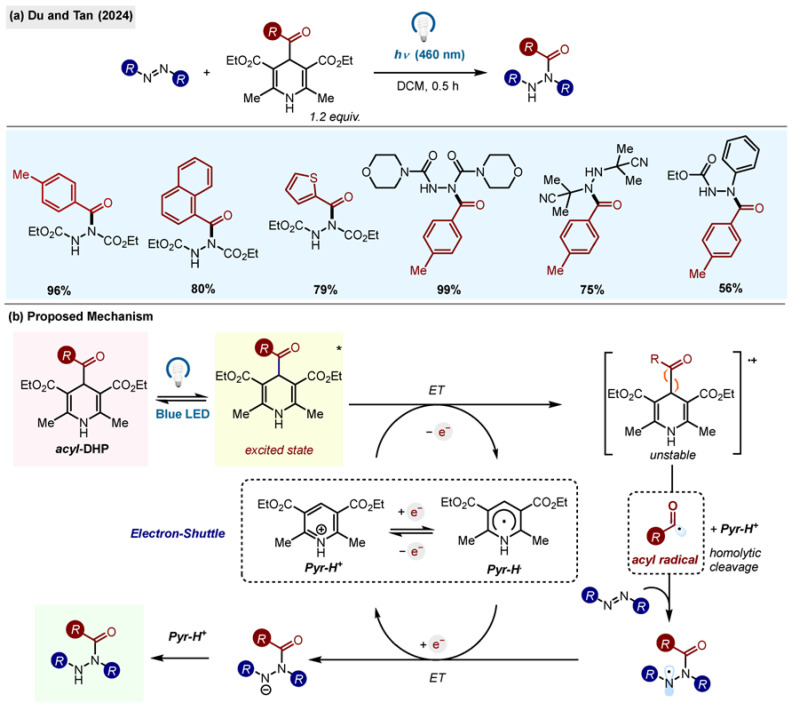
(**a**) Hydroacylation of azodicarboxylic acid derivatives by Du and Tan (2024) [51]. (**b**) Proposed mechanism.

The Tan group treated diethyl azodicarboxylate (DEAD) with acyl-1,4-DHP under blue light, forming the desired adduct in a 95% yield (Scheme 20). Various aromatic radical precursors were probed with both electron-donating and electron-withdrawing groups, affording the desired products in moderate to good yields (56–99%). However, reagents consisting of aliphatic groups failed to deliver the expected product, possibly due to the low stability of the corresponding aliphatic acyl radical species. Dipiperidines, dimorpholides and diazene products all proved to be attainable. Unsymmetrical azo-compounds were also synthesized at moderate yields.

The Tan group also came to the conclusion that the reaction starts with the single-electron oxidation of the acyl-1,4-DHP, followed by fragmentation to release the acyl radical. It is also postulated that **Pyr-H^+^** acts as an electron shuttle (Scheme 20b).

### 2.8. Radical Trifluoromethylthiolation and Trifluoromethylselenolation

**Scheme 21:**
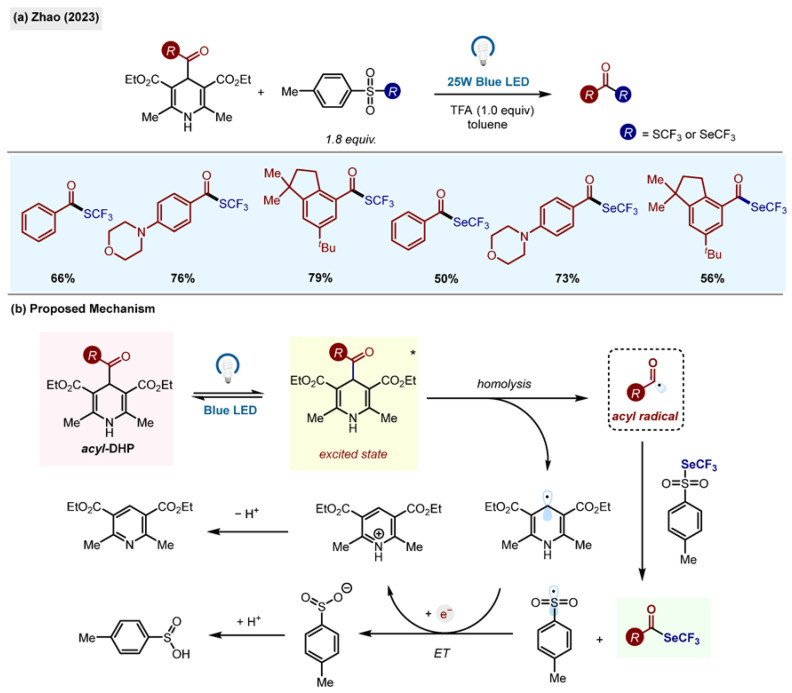
(**a**) Addition of SCF_3_ and SeCF_3_ to acyl radicals by Zhao (2023) [52]. (**b**) Proposed mechanism.

SCF_3_ and SeCF_3_ groups in organic molecules have attracted major interest from both academic and industrial faculties in the past decade due to their high lipophilicity and cell membrane permeability. Zhao et al. presented a visible light-promoted methodology towards trifluoromethylthiolation and trifluoroselenolation of acyl radicals (Scheme 21) [52]. The optimized conditions found included toluene as a solvent, trifluoroacetic acid and blue LED under nitrogen at room temperature.

The scope of the reaction included a vast array of acyl-1,4-DHPs as substrates, all resulting in the desired products in moderate to good yields. 4-(alkylcarbonyl)-1,4-DHP as a substrate also proved fruitful with the competitive C(sp_3_)-SCF_3_ bond formation via decarbonylation of the aliphatic acyl radical, which was not observed. The reaction using its selenium counterpart also yielded the desired product in good yields. The generality of the trifluoromethylselenolation reaction was investigated using a wide range of acyl-1,4-DHPs, again showing good tolerance and yields.

The proposed mechanism begins with the excitation of acyl-1,4-DHP using visible light irradiation followed by the homolytic cleavage of the excited species resulting in the formation of the DHP radical and an acyl radical.

The acyl radical is trapped by the sulfonothioate or the selenoate, yielding the product and the sulfone/selenone radical which is transformed to its cation counterpart. Finally, the cation is reduced to the sulfite/selenite via single-electron transfer with the DHP radical. The C-SCF_3_/C-SeCF_3_ is scalable to a gram scale, indicating the practical applicability of the transformation.

**Scheme 22:**
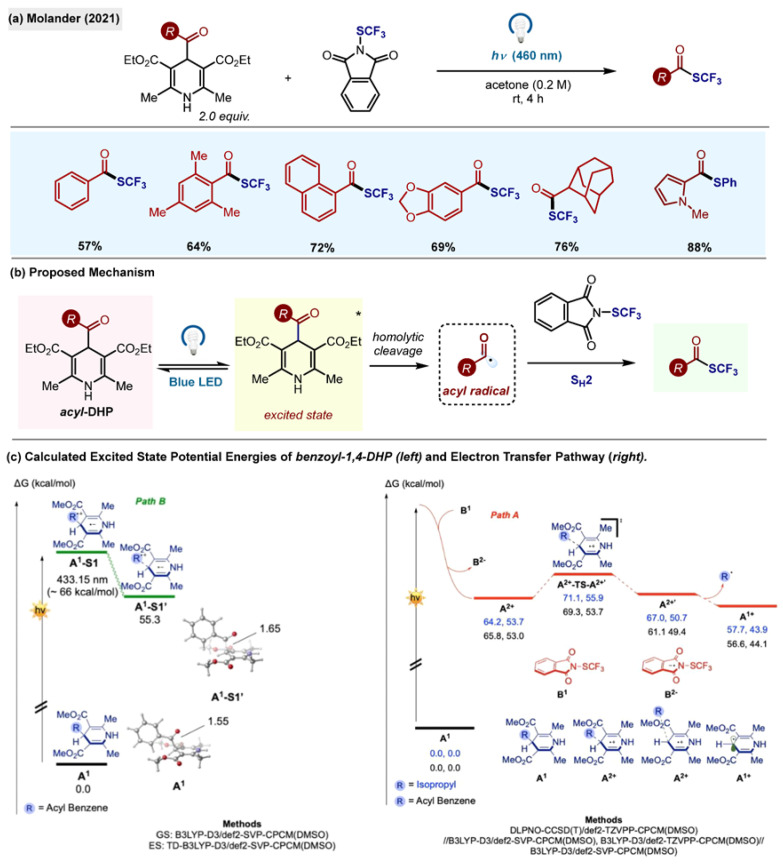
(**a**) Trifluoromethylthiolation of acyl radicals by Molander (2021) [32]. (**b**) Proposed Mechanism. (**c**) Potential energy surfaces: for the excitation of benzoyl-1,4-DHP **1** (**left**) and acyl radical generation mediated by electron transfer between benzoyl-1,4-DHP and *N*-(trifluoromethylthio)phthalimide (**right**). Labelled bonds in Ångstroms. Reproduced from [32]).

Trifluoromethylthiolation of acyl radicals was reported by Molander, utilizing acyl-1,4-DHP as a radical source and *N*-(trifluoromethylthio)phthalimide as an acceptor (Scheme 22a) [32]. The reaction enabled the formation of a diverse set of trifluoromethylthiolated aldehydes in high yields upon the direct excitation of acyl-1,4-DHPs with a visible light. It was also found that the SCN and SPh groups could be acylated in a similar manner. The reaction mixture was kept open to air and the method was scalable.

The reaction mechanism was studied in detail by quantum mechanical calculations (Scheme 22b,c). The authors proposed the homolytic cleavage of the C-C σ-bond of the acyl-1,4-DHP upon excitation with visible light. This theory was supported by computational findings of the elongation of the C-C σ-bond in the excited state of benzoyl-1,4-DHP **1** from 1.55 to 1.65 Å (Scheme 22c). Another possibility was the activity of the observed electron donor–acceptor (EDA) complex between *N*-(trifluoromethylthio)phthalimide and acyl-1,4,-DHP, evidenced by the hypsochromic shift from 433 nm to 418 nm. However, EDA complexation was found not to be necessary for the generation of acyl radicals in this case.

### 2.9. Photoinduced Charge Transfer Complexes (EDA)

An association between electron-rich and electron-deficient molecules in some cases lead to the formation of electron donor–acceptor complexes (EDA), also known as charge transfer complexes [53]. These aggregates exhibit special properties, including a red shift in the UV/Vis absorbance spectrum. The energy of this new band quite often lies in the visible region and its irradiation leads to an electron transfer from the donor molecule to the acceptor. Most commonly, the EDA complex relaxes to the ground state by back electron transfer, but if the acceptor molecule bears a leaving group, this process may lead to fragmentation and further transformations, unlocking new modes of reactivity.

The advantage of the EDA chemistry lies in bypassing multiple problems and complications related to the photochemical activation of substrates, including the necessity of using photocatalysts or photosensitizers, high redox potentials, stabilities, selectivity issues and sensitivities, amongst other issues.

**Scheme 23:**
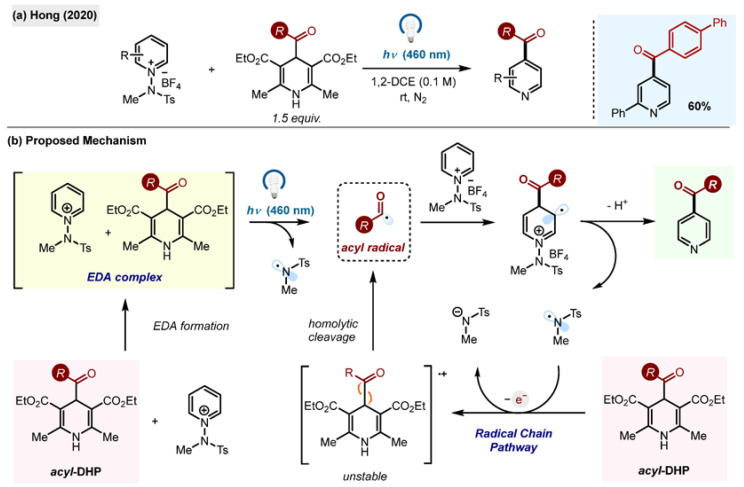
(**a**) Acyl-1,4-DHP as donors in EDA complex chemistry: 4-selective functionalization of pyridines by Hong (2020) [54]. (**b**) Proposed mechanism.

It was found that acyl-1,4-DHPs, which are electron-rich compounds, can form charge transfer complexes with certain electron-poor species, leading to new chemical reactions. The EDA formation between acyl-1,4-DHP and *N*-amidopyridinium salts was observed and exploited by the research group of Hong for a C4-selective acylation of pyridines (Scheme 23a) [54]. Classical, non-EDA-triggered acyl radical additions to these *N*-heteroarenes produce mixtures of C2-, C4- and C6- functionalized products.

Here, the application of the charge transfer mechanism enabled the switching of the selectivity towards the C4 position exclusively. The reaction was found to be general for alkyl, acyl and carbamoyl radicals derived from 1,4-DHPs. The proposed mechanism relies on an ET from an acyl-1,4-DHP to the *N*-aminopyridinium salt, followed by the release of radicals and initiation of a radical chain pathway. The evidence for the radical chain was provided by the quantum yield measurements (*ϕ* = 16). The method was applied to C4-selective late-stage functionalization of pharmaceutically relevant substrates.

### 2.10. Other Applications in Organic Synthesis

**Scheme 24:**
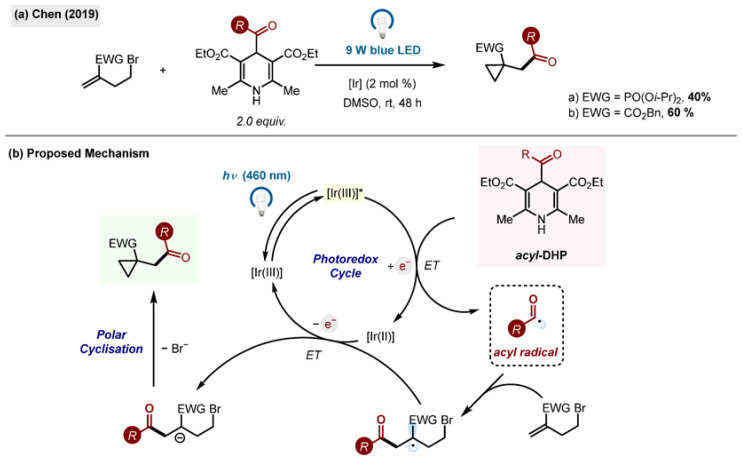
(**a**) Radical–polar crossover by Chen (2019) [55]. (**b**) Proposed mechanism.

**Scheme 25:**
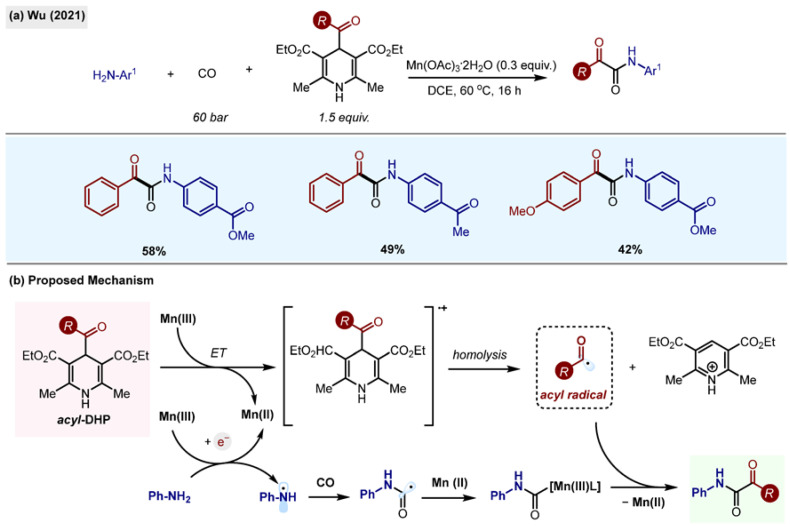
(**a**) Double carbonylation of anilines by Wu (2021) [56]. (**b**) Proposed mechanism.

**Scheme 26:**
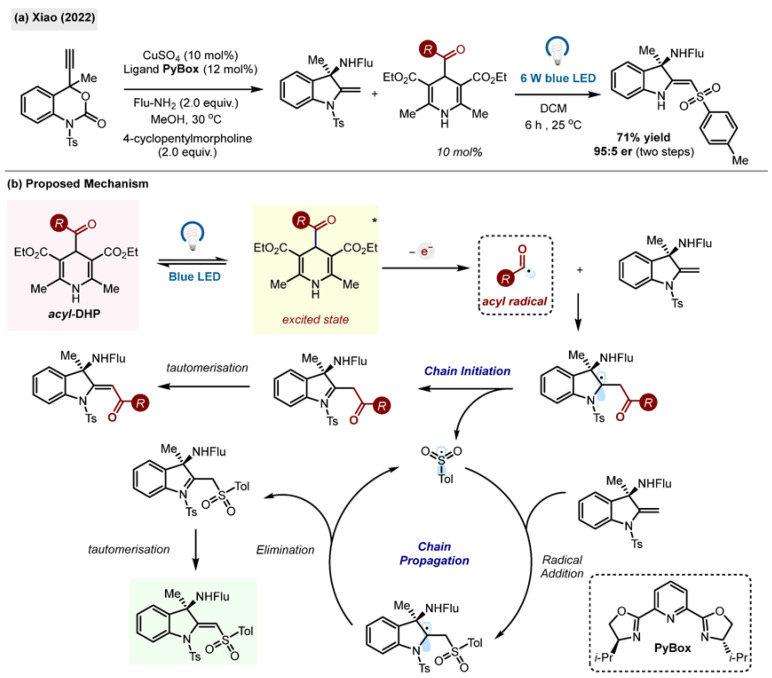
(**a**) Acyl-1,4-DHP triggered sulfonyl migration by Xiao (2022) [58]. (**b**) Proposed mechanism.

**Scheme 27:**
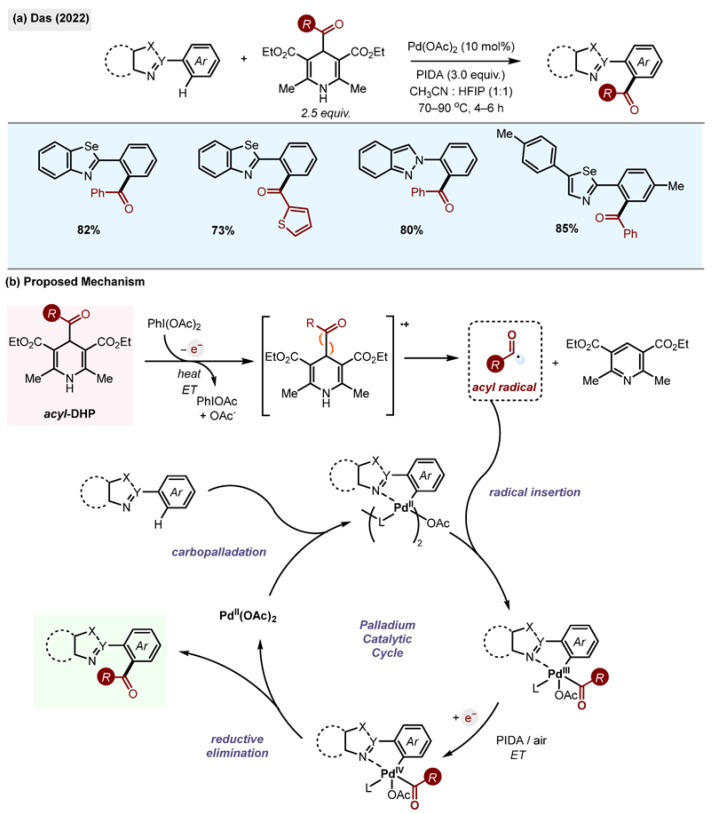
(**a**) Palladium-catalyzed *o*-acylation of azoles by Das (2022) [59]. (**b**) Proposed mechanism.

**Scheme 28:**
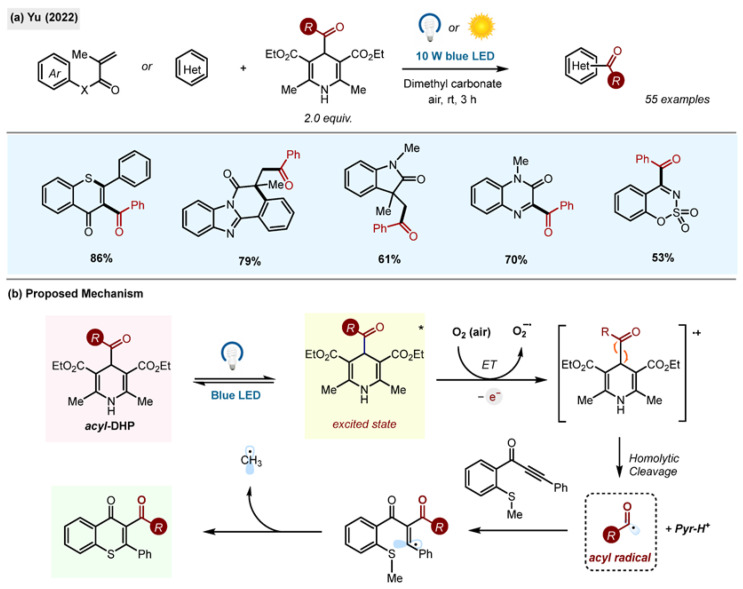
(**a**) Green radical cascade reaction for the synthesis of acylated heterocycles by Yu (2022) [28]. (**b**) Proposed mechanism.

**Scheme 29:**
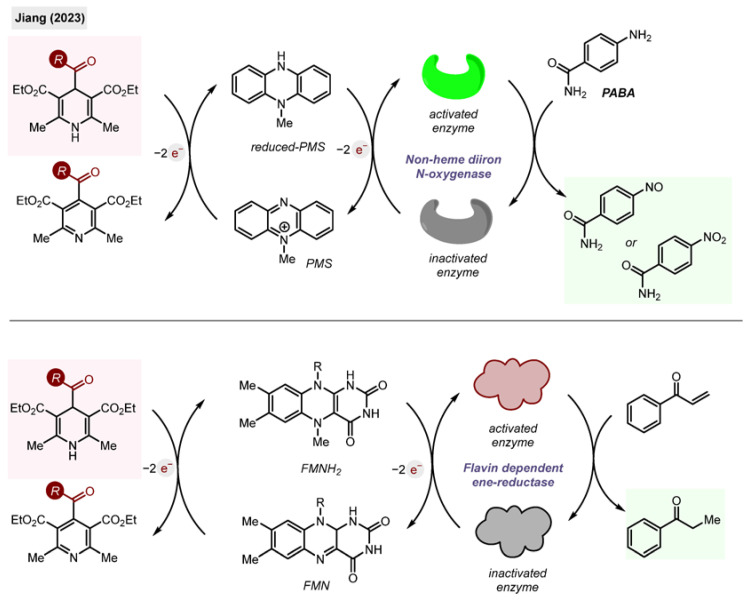
Acyl-1,4-DHP as an electron source and NADH replacement in biosynthesis by Jiang (2023) [60].

Acyl-1,4-DHPs have also found applications in radical–polar crossover reactions, in forming cyclopropanes (Scheme 24), in transition metal double carbonylation of anilines (Scheme 25), as radical initiators for a stereospecific sulfonyl migration reaction (Scheme 26), as an acyl source for Pd(II) *o*-functionalisation of azoles (Scheme 27) and as a key reagent for the green construction of heterocycles (Scheme 28). Finally, acyl-1,4-DHPs were adopted for enzymatic reactions as more economical NADH analogues (Scheme 29). 

#### 2.10.1. Radical–Polar Crossover Reactions

Cyclopropanes are an important structural motif in many natural products and pharmaceuticals (Scheme 24). The research put forward by the group of Chen outlines a photoredox-catalyzed allylation/cyclopropanation cascade reaction via a radical addition and polar cyclization, presenting an attractive alternative to the well-established stepwise formation of cyclopropanes [55]. Using vinylphosphonate and *n*-octylsilicate for the initial model reaction, the conditions were optimized, employing Ir[dF(CF_3_)ppy]_2_(dtbbpy)PF_6_ as the photocatalyst in conjunction with blue light in DMSO, resulting in an 82% yield of cyclopropane adduct. Acyl-1,4-DHPs were employed to further probe the reaction, resulting in the generation of the acyl radical in place of the alkyl radicals from the silicate.

Mechanistic investigations were conducted using TEMPO. It was confirmed that the reaction proceeds via a radical pathway. A plausible mechanism for the radical–polar crossover reaction was put forward with the single-electron oxidation of the acyl-1,4-DHP by the photocatalyst forming the acyl radical which can add to the 1,1-disubstituted alkene. This forms a new carbon radical which undergoes single-electron reduction, forming the intermediate which is reduced and forming the final 3-*exo-tet* cyclized cyclopropane.

#### 2.10.2. Double Carbonylation of Anilines

Synthesis of α-ketoamides by Mn(OAc)_3_ promoted double carbonylation of anilines with acyl-1,4-DHP, which was achieved by the group of Wu (Scheme 25) [56]. α-Ketoamides are important structural motifs among natural products and APIs. They are also used as probes for H_2_O_2_ intracellular visualization [57]. The protocol relies on the oxidation of trifluoroborate or DHP radical precursors and anilines by Mn(III) salt, followed by CO incorporation, radical trapping by the Mn atom and reductive elimination to form double-carbonylated products. Acyl radicals are formed by the carbonylation of alkyl radicals with CO, or directly, from acyl-1,4-DHP.

The process is operationally simple, and inexpensive Mn(III) salt is used as an internal oxidant and promoter of the generation of radical species. The scope of the reaction is broad, and the method tolerates a variety of functional groups such as iodides, which would otherwise be reactive under palladium cross-coupling conditions. 

#### 2.10.3. Stereospecific Sulfonyl Migration

Acyl-1,4-DHPs can also act as radical initiators, equivalent to CF_3_ radicals in Cu-catalyzed asymmetric cyclisation and sulfonyl migration (Scheme 26) [58]. Through this unusual transformation, new aminoindolines were prepared with a high degree of stereoselectivity by the research group of Xiao.

The authors also reported unexpected sulfonyl radical migration with retained enantiopurity. The synthesized molecules were found to exhibit anti-tumour properties. The acyl radicals, generated by direct irradiation of benzoyl-1,4-DHP **1,** acted as efficient initiators for the radical sulfonyl migration step.

#### 2.10.4. Pd(II)-Catalyzed Ortho-Acylation of Azoles

Transition metal-powered regioselective functionalization of C-H bonds has been an area of increasing interest for the formation of functionalized organic molecules. In regioselective C-H functionalization, directing groups such as sp*^2^*-*N*-containing heterocycles have played a crucial role. However, selenium-containing compounds have rarely been investigated due to their subpar chelation with transition metals.

Studies by Das et al., utilize 1,4-DHPs as sources of radicals in the presence of Pd(OAc)_2_ and iodobenzene diacetate (PIDA) as an oxidant for the ortho functionalisation of azoles (Scheme 27) [59]. The substrate scope of the reaction was probed with a variety of substituted selenazoles, isoselenazoles and indazole motifs, forming the benzoylated product in good yields. The reaction was attempted with various acyl-1,4-DHP reagents, all yielding the desired product in good yields.

A mechanism for the reaction was proposed whereby a single-electron oxidation of the 1,4-DHP by PIDA takes place upon the application of heat. This forms a carbon-centred radical along with an iodine radical with the release of pyridine as a side product.

It is suggested that the azole coordinates with the palladium, followed by the insertion of the carbon-centred radical into the palladium, forming a trivalent intermediate which undergoes single-electron oxidation by PIDA to a Pd(IV) complex, which subsequently eliminates the formation of the desired *o*-acylated product and regenerates the palladium catalyst. To demonstrate the utility of the methodology, the above reaction was performed at a one-gram scale with good yields.

#### 2.10.5. Green Radical Cascade Reactions

The synthetic potential of acyl-1,4-DHPs in the synthesis of acylated heterocycles via radical cascade reactions was explored by the Yu research group (Scheme 28) [28]. The authors used acyl-1,4-DHPs as universal acylating reagents. Blue light activation in dimethyl carbonate, a green solvent, enabled the formation of a diverse set of products, including thioflavones, benzimidazo[2,1-a]isoquinolin-6(5H)-ones, indolo[2,1-a]isoquinolin-6(5H)ones, quaternary 3,3-dialkyl 2-oxindoles, quinoxalin-(1H)-ones, benzo[e][1–3]oxathiazine 2,2-dioxides and others. The method operated under mild conditions, at room temperature and open to air. The reaction was also found scalable to 1 g. It was also possible to activate the reaction in high efficiency by direct sunlight.

The proposed reaction mechanism involves direct excitation of acyl-1,4-DHP, which transfers an electron to triplet oxygen in air (Scheme 28b). The feasibility of this step was supported by Stern–Volmer quenching experiments, indicating that O_2_ was an effective quencher of the excited-state DHP. The study of the reaction sensitivity and reproducibility revealed that the only factor affecting the reproducibility was the light intensity, with less intense light producing less product. Other factors, such as concentration, water content, oxygen content, temperature and scale, had no effect on the reaction outcome, revealing its high tolerance. This method avoided the use of catalysts, external oxidants or standard organic solvents.

#### 2.10.6. Biosynthesis

Finally, the acyl-1,4-DHPs found applications for biosynthesis as NADH analogues [60]. DHPs, including **1,** were tested as cost-efficient substitutions for NADH co-factors in enzymatic processes.

The group of Jiang found that DHPs are efficient mimickers of NADH in four enzymatic redox transformations, using non-heme diiron N-oxygenases and flavin dependent ene-reductases (Scheme 29). The role of the DHP was to provide electrons to reduce PMS and FMN for subsequent transformations.

## 3. Conclusions

In its short history, acyl-1,4-DHP has emerged as a convenient and powerful reagent of choice for a variety of synthetic applications, with acylation via acyl radical addition to various radical acceptors being the most prominent. Keto groups, introduced upon acylation, are among the most prevalent groups in organic chemistry. This diverse set of reactivities is owed to the ability of 1,4-DHPs to act as a single-electron reductant in the ground and excited state, forming unstable radical cation **RC** upon oxidation. The experimental evidence supports the theory that it is the decomposition of the **RC** that releases acyl radicals, which are used in synthesis. Acyl-1,4-DHP is therefore a dual reagent—a source of electrons and acyl radicals.

Acyl-1,4-DHPs are bench-stable, easy to make reagents derived from glyoxal chemical feedstock. Their use is operationally simple, utilizing visible light or direct sunlight, electrochemistry or inexpensive chemical oxidants for their activation, by-passing the need for photocatalysts, photosensitizers or expensive transition metals. They have also been found to engage in the chemistry of EDA complexes, unlocking new patterns of reactivity. Not all desired acyl radicals can be formed from acyl-1,4-DHPs, however. The clear limitation of the current state of the art is the lack of procedures for the preparation of certain important chemical motifs, including benzyl and linear alkyl chains attached to the acyl moiety.

## Data Availability

Not applicable.

## References

[1] Hantzsch A. (1881). Condensationsprodukte aus Aldehydammoniak und ketonartigen Verbindungen. Chem. Ber..

[2] Nelson D.L., Cox M.M. (2021). Leininger Principles of Biochemistry.

[3] Eisner U., Kuthan J. (1972). Chemistry of dihydropyridines. Chem. Rev..

[4] Stout D.M., Meyers A.I. (1989). Recent advances in the chemistry of dihydropyridines. Chem. Rev..

[5] Lavilla R. (2002). Recent developments in the chemistry of dihydropyridines. J. Chem. Soc. Perkin Trans..

[6] Parthiban A., Makam P. (2022). 1,4-Dihydropyridine: Synthetic advances, medicinal and insecticidal properties. RSC Adv..

[7] Edraki N., Mehdipour A.R., Khoshneviszadeh M., Miri R. (2009). Dihydropyridines: Evaluation of their current and future pharmacological applications. Drug Discov. Today.

[8] Yang J.W., Fonseca M.T.H., List B. (2004). A Metal-Free Transfer Hydrogenation: Organocatalytic Conjugate Reduction of α,β-Unsaturated Aldehydes. Angew. Chem. Int. Ed..

[9] Ouellet S.G., Tuttle J.B., MacMillan D.W.C. (2005). Enantioselective Organocatalytic Hydride Reduction. J. Am. Chem. Soc..

[10] Wu L.-M., Chen W., Liu Z.-L. (2001). Stable free radicals generated during the oxidation of 4-alkyl Hantzsch 1,4-dihydropyridines with nitrosonium—EPR evidence. Res. Chem. Intermed..

[11] Nakajima K., Nojima S., Sakata K., Nishibayashi Y. (2016). Visible-Light-Mediated Aromatic Substitution Reactions of Cyanoarenes with 4-Alkyl-1,4-dihydropyridines through Double Carbon–Carbon Bond Cleavage. ChemCatChem.

[12] Gutiérrez-Bonet A., Tellis J.C., Matsui J.K., Vara B.A., Molander G.A. (2016). 1,4-Dihydropyridines as Alkyl Radical Precursors: Introducing the Aldehyde Feedstock to Nickel/Photoredox Dual Catalysis. ACS Catal..

[13] Kurz J.L., Hutton R., Westheimer F.H. (1961). The Photochemical Reduction of Bromotrichloromethane by Derivatives of 1,4-Dihydropyridine. J. Am. Chem. Soc..

[14] Buzetti L., Prieto A., Roy S.R., Melchiorre P. (2017). Radical-Based C−C Bond-Forming Processes Enabled by the Photoexcitation of 4-Alkyl-1,4-dihydropyridines. Angew. Chem. Int. Ed..

[15] Goti G., Bieszczad B., Vega-Peñaloza A., Melchiorre P. (2019). Stereocontrolled Synthesis of 1,4-Dicarbonyl Compounds by Photochemical Organocatalytic Acyl Radical Addition to Enals. Angew. Chem. Int. Ed..

[16] Zheng C., You S.-L. (2012). Transfer hydrogenation with Hantzsch esters and related organic hydride donors. Chem. Soc. Rev..

[17] Phillips A.M.F., Pombeiro A.J.L. (2023). Applications of Hantzsch Esters in Organocatalytic Enantioselective Synthesis. Catalysts.

[18] Singh S., Sharma V.K., Gill S., Sahota R.I.K. (1985). Reduction of imines by NADH models. J. Chem. Soc. Perkin Trans. 1.

[19] Wang P.-Z., Chen J.-R., Xiao W.-J. (2019). Hantzsch esters: An emerging versatile class of reagents in photoredox catalyzed organic synthesis. Org. Biomol. Chem..

[20] Yedase G.S., Venugopal S., Arya P., Yatham V.R. (2022). Catalyst-free Hantzsch Ester-mediated Organic Transformations Driven by Visible light. Asian J. Org. Chem..

[21] Shengqing Y., Jie W. (2019). 4-Substituted Hantzsch Esters as Alkylation Reagents in Organic Synthesis. Acta Chim. Sin..

[22] Jia X., Yu L., Huo C., Wang Y., Liu J., Wang X. (2014). Catalytic aromatization of 1,4-dihydropyridines by radical cation salt prompted aerobic oxidation. Tetrahedron Lett..

[23] Alandini N., Buzetti L., Favi G., Schulte T., Candish L., Collins K.D., Melchiorre P. (2020). Amide Synthesis by Nickel/Photoredox-Catalysed Direct Carbamoylation of (Hetero)Aryl Bromides. Angew. Chem. Int. Ed..

[24] Chatgilialoglu C., Crich D., Komatsu M., Ryu I. (1999). Chemistry of Acyl Radicals. Chem. Rev..

[25] Luo X., Wang P. (2021). Ynonylation of Acyl Radicals by Electroinduced Homolysis of 4-Acyl-1,4-dihydropyridines. Org. Lett..

[26] Liu L., Jiang P., Liu Y., Du H., Tan J. (2020). Direct radical alkylation and acylation of 2H-indazoles using substituted Hantzsch esters as radical reservoirs. Org. Chem. Front..

[27] Bieszczad B., Perego L.A., Melchiorre P. (2019). Photochemical C−H Hydroxyalkylation of Quinolines and Isoquinolines. Angew. Chem. Int. Ed..

[28] Zeng F.-L., Xie K.-C., Liu Y.-T., Wang H., Yin P.-C., Qu L.-B., Chen X.-L., Yu B. (2022). Visible-light-promoted catalyst-/additive-free synthesis of aroylated heterocycles in a sustainable solvent. Green Chem..

[29] Mandal R.D., Saha M., Das D., Das A.R. (2023). Electrochemically Enabled C4–H and C3–H Functionalization of 2-Phenyl Quinazoline and Quinoxaline through Dehydrogenative C–H/C–H, C–H/P–H, and C–H/O–H Cross-Coupling. J. Org. Chem..

[30] Shen G.-B., Xie L., Yu H.Y., Liu J., Fu Y.-H., Yan M. (2020). Theoretical investigation on the nature of 4-substituted Hantzsch esters as alkylation agents. RSC Adv..

[31] Shen G.-B., Xie L., Wang Y.-X., Gong T.-Y., Wang B.-Y., Hu Y.-H., Fu Y.-H., Yan M. (2021). Quantitative Estimation of the Hydrogen-Atom-Donating Ability of 4-Substituted Hantzsch Ester Radical Cations. ACS Omega.

[32] Lipp A., Badir S.O., Dykstra R., Gutierrez O., Molander G.A. (2021). Catalyst-Free Decarbonylative Trifluoromethylthiolation Enabled by Electron Donor-Acceptor Complex Photoactivation. Adv. Synth. Catal..

[33] Mondal S., Dumur F., Gimes D., Sibi M.P., Bertrand M.P., Nechab M. (2022). Enantioselective Radical Reactions Using Chiral Catalysts. Chem. Rev..

[34] Zhang K., Lu L.-Q., Jia Y., Wang Y., Lu F.-D., Pan F., Xiao W.-J. (2019). Exploration of a Chiral Cobalt Catalyst for Visible-Light-Induced Enantioselective Radical Conjugate Addition. Angew. Chem. Int. Ed..

[35] Duncton M.A.J. (2011). Minisci reactions: Versatile C-H functionalisations for medicinal chemists. Med. Chem. Commun..

[36] Proctor R.S.J., Phipps R.J. (2019). Recent Advances in Minisci-Type Reactions. Angew. Chem. Int. Ed..

[37] Byun Y., Moon J., An W., Mishra N.K., Kim H.S., Ghosh P., Kim I.S. (2021). Transition-Metal-Free Alkylation and Acylation of Benzoxazinones with 1,4-Dihydropyridines. J. Org. Chem..

[38] Tariq S., Somakala K., Amir M. (2018). Quinoxaline: An insight into the recent pharmacological advances. Eur. J. Med. Chem..

[39] Ghosh P., Kwon N.Y., Byun Y., Mishra N.K., Park J.S., Kim I.S. (2022). Cobalt(II)-Catalyzed C–H Alkylation of N-Heterocycles with 1,4-Dihydropyridines. ACS Catal..

[40] Zhao X., Li B., Xia W. (2020). Visible-Light-Promoted Photocatalyst-Free Hydroacylation and Diacylation of Alkenes Tuned by NiCl_2_·DME. Org. Lett..

[41] Liu L., Deng Z., Xu K., Jiang P., Du H., Tan J. (2021). Access to Deuterated Unnatural α-Amino Acids and Peptides by Photochemical Acyl Radical Addition. Org. Lett..

[42] Jin S., Zhang L. (2022). Expedient access to *N*-alkylphthalimides via redox-neutral photocatalysed Giese-type reactions. Org. Biomol. Chem..

[43] Pálvölgyi Á.M., Ehrschwendtner F., Schnürch M., Bica-Schröder K. (2022). Photocatalyst-free hydroacylations of electron-poor alkenes and enones under visible-light irradiation. Org. Biomol. Chem..

[44] Zhang Z., Wang J., Yu C., Tan J., Du H., Chen N. (2024). Visible-Light-Induced Acylative Pyridylation of Styrenes. Org. Lett..

[45] Kobayashi S., Ishitani H. (1999). Catalytic Enantioselective Addition to Imines. Chem. Rev..

[46] Choudhury L.H., Parvin T. (2011). Recent advances in the chemistry of imine-based multicomponent reactions (MCRs). Tetrahedron.

[47] Uchikura T., Kamiyama N., Mouri T., Akiyama T. (2022). Visible-Light-Driven Enantioselective Radical Addition to Imines Enabled by the Excitation of a Chiral Phosphoric Acid–Imine Complex. ACS Catal..

[48] Li J., Phetcharawetch J., Qi M., Kyne S.H., Kuhakarn C., Zhong B., Chan P.W.H. (2023). Organocatalytic alkylation and photoorganocatalyst-free acylation of azomethine imines by Hantzsch esters under blue LED light. New. J. Chem..

[49] Song X., Zhang Y., Li Y., Zhao X., Yin Y., Ban X., Jiang Z. (2023). Catalytic Asymmetric Synthesis of Azaarene-Functionalized Tertiary Amines and α-Amino Acid Derivatives from *E*/*Z*-Ketimine Mixtures via Enantioselective Radical Coupling. ACS Catal..

[50] Zhong Z., Wu H., Chen X., Luo Y., Yang L., Feng X., Liu X. (2024). Visible-Light-Promoted Enantioselective Acylation and Alkylation of Aldimines Enabled by 9-Fluorenone Electron-Shuttle Catalysis. J. Am. Chem. Soc..

[51] Liu L., Wang J., Feng X., Xu K., Liu W., Peng X., Du H., Tan J. (2024). Visible-Light-Mediated Photocatalyst-Free Hydroacylation of Azodicarboxylic Acid Derivatives with 4-Acyl-1,4-dihydropyridines. Chin. J. Chem..

[52] Shan X., Wang X., Chen E., Liu J., Zhao X. (2023). Visible-Light-Promoted Trifluoromethylthiolation and Trifluoromethylselenolation of 1,4-Dihydropyridines. J. Org. Chem..

[53] Crisenza G.E.M., Mazzarella D., Melchiorre P. (2020). Synthetic Methods Driven by the Photoactivity of Electron Donor–Acceptor Complexes. J. Am. Chem. Soc..

[54] Kim I., Park S., Hong S. (2020). Functionalization of Pyridinium Derivatives with 1,4-Dihydropyridines Enabled by Photoinduced Charge Transfer. Org. Lett..

[55] Luo W., Yang Y., Fang Y., Zhang X., Jin X., Zhao G., Zhang L., Li Y., Zhou W., Xia T. (2019). Photoredox-Catalyzed Cyclopropanation of 1,1-Disubstituted Alkenes via Radical-Polar Crossover Process. Adv. Synth. Catal..

[56] Chen B., Kuai C.-S., Xu J.-X., Wu X.-F. (2022). Manganese(III)-Promoted Double Carbonylation of Anilines Toward α-Ketoamides Synthesis. Adv. Synth. Catal..

[57] Gao P., Pan W., Li N., Tang B. (2019). Fluorescent probes for organelle-targeted bioactive species imaging. Chem. Sci..

[58] Wang B.-C., Fan T., Xiong F.-Y., Chen P., Fang K.-X., Tan Y., Lu L.-Q., Xiao W.-J. (2022). De Novo Construction of Chiral Aminoindolines by Cu-Catalyzed Asymmetric Cyclization and Subsequent Discovery of an Unexpected Sulfonyl Migration. J. Am. Chem. Soc..

[59] Sarkar A., Saha M., Das A.R., Banerjee A., Majumder R., Bandyopadhyay D. (2022). Hypervalent iodine mediated Pd(II)-catalyzed ortho-C(sp2−H) functionalization of azoles deciphering Hantzsch ester and malononitrile as the functional group surrogates. ChemistrySelect.

[60] Guo Y.-Y., Tian Z.-H., Han Y.-C., Ma D., Shao T., Jiang Z. (2023). Hantzsch Ester as Efficient and Economical NAD(P)H Mimic for In Vitro Bioredox Reactions. Chem. Eur. J..

